# Signatures of cross-modal alignment in children’s early concepts

**DOI:** 10.1073/pnas.2309688120

**Published:** 2023-10-11

**Authors:** Kaarina Aho, Brett D. Roads, Bradley C. Love

**Affiliations:** ^a^Department of Experimental Psychology, University College London, London WC1H 0AP, United Kingdom; ^b^The Alan Turing Institute, London NW1 2DB, United Kingdom

**Keywords:** learning, alignment, multimodal, asynchronous

## Abstract

Whether supervised or unsupervised, human and machine learning is usually characterized as event-based. However, learning may also proceed by systems alignment in which mappings are inferred between entire systems, such as visual and linguistic systems. Systems alignment is possible because items that share similar visual contexts, such as a car and a truck, will also tend to share similar linguistic contexts. Because of the mirrored similarity relationships across systems, the visual and linguistic systems can be aligned at some later time absent either input. In a series of simulation studies, we considered whether children’s early concepts support systems alignment. We found that children’s early concepts are close to optimal for inferring novel concepts through systems alignment, enabling agents to correctly infer more than 85% of visual–word mappings absent supervision. One possible explanation for why children’s early concepts support systems alignment is that they are distinguished structurally by their dense semantic neighborhoods. Artificial agents using these structural features to select concepts proved highly effective, both in environments mirroring children’s conceptual world and those that exclude the concepts that children commonly acquire. For children, systems alignment and event-based learning likely complement one another. Likewise, artificial systems can benefit from incorporating these developmental principles.

## Significance

Can people relate patterns of visual and linguistic experience to one another when they do not co-occur? Remarkably, people can and do use this information to make inferences about novel concepts. What enables this alignment is that items that share similar visual contexts, such as a car and a truck, also tend to share similar linguistic contexts. These mirrored similarity relationships make it possible to align the systems, which in turn makes it possible to infer the names of objects absent supervision. Children’s early concepts form dense networks, which are particularly well suited for aligning systems. Artificial agents that incorporate these developmental principles outperform other models.

Learning can occur in many ways but is almost always cast as event-based learning. Event-based learning can take multiple forms—such as supervised, semi-supervised, weakly supervised, and unsupervised learning (1)—but assumes that all of the necessary information for learning occurs within a narrow time window. Consider a supervised learning event where a child’s caregiver points to a dog and says “dog.” In this example, the visual input and verbal label both occur at almost the same time. Similarly, a weakly- or semi-supervised event may occur when a child overhears a conversation between two adults. While temporal proximity is a strong clue during learning, it is not a panacea.

Even a direct labeling event is ambiguous because labeling is underconstrained (2–5). Nevertheless, infants learn from indirect word exposure absent direct labeling, either through overhearing or interactions not intended as learning events (6–12). They can also resolve ambiguous labels by combining information across different events, i.e., cross-situational statistics (13). Children’s use of co-occurrence information to infer meaning from natural language appears somewhat analogous to how self-supervised machine learning systems use structure in the data as a supervisory signal (14–20). Infants are sensitive to co-occurrences in language from a young age (21), and semantic information can be derived from the co-occurrence statistics in child-directed speech (22). All of these learning feats support the idea that children have a profound ability to infer conceptual relationships, even when those relationships are not directly observed.

**Fig. 1.**
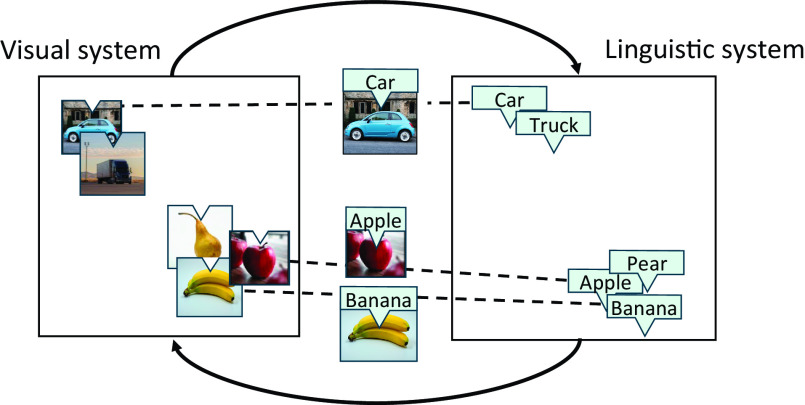
Inferring visual–word mappings through systems alignment. Dashed lines between systems represent known visual–word mappings, or concepts. Here, the agent knows the visual–word mappings for apple, banana, and car, but not the mappings for pear and truck. Based on the similarity relationships within the systems, the agent could correctly map pear and truck when presented with the two objects. Having heard the words “truck” and “pear” being used in context, they would know that “truck” occurs in similar contexts to “car,” whereas “pear” occurs in similar contexts to “apple” and “banana.” The visual contexts pattern in much the same way (e.g., both cars and trucks are found on roads). These repeating similarity relations across systems make it possible to correctly infer which visual object is the pear and which is the truck. This type of forced choice between two pairs of visual objects and words was used to evaluate artificial agents in our simulations.

Event-based learning is unquestionably an effective route for human learning, but we argue that people use an additional mode of learning that is distinct from event-based learning. In this work, we present evidence that information beyond individual events could be exploited to align systems (e.g., to discover new mappings between visual and linguistic systems), which we refer to as systems alignment. We define a system as the similarity relations between items in a representational space. Systems alignment is the use of similarity relations that are mirrored across multiple systems to perform a cross-system mapping (Fig. 1). While systems alignment is a general mechanism, the current study is concerned with understanding the value of systems alignment for agents learning to map between visual and linguistic systems.

Unlike event-based learning that relies on temporally proximate information, such as seeing a dog and hearing “dog” as in the above example, systems alignment can be asynchronous such that information is acquired at different times in the visual and linguistic systems and can be aligned at some later time absent either input. This is a key distinction between this mechanism and previous multimodal learning approaches (23, 24). The asynchronous nature of alignment may help explain how label-referent mappings are learned despite their relatively infrequent co-occurrence in children’s sensory input: Recordings obtained from cameras mounted on children’s heads in naturalistic environments reveal that the simultaneous experience of visual object and its corresponding label is rare, with absent objects frequently being referenced, and visual objects not being named (25). Further, 60 to 70% of concrete nouns in child-directed speech are not in reference to the current environment or activity (26).

**Fig. 2.**
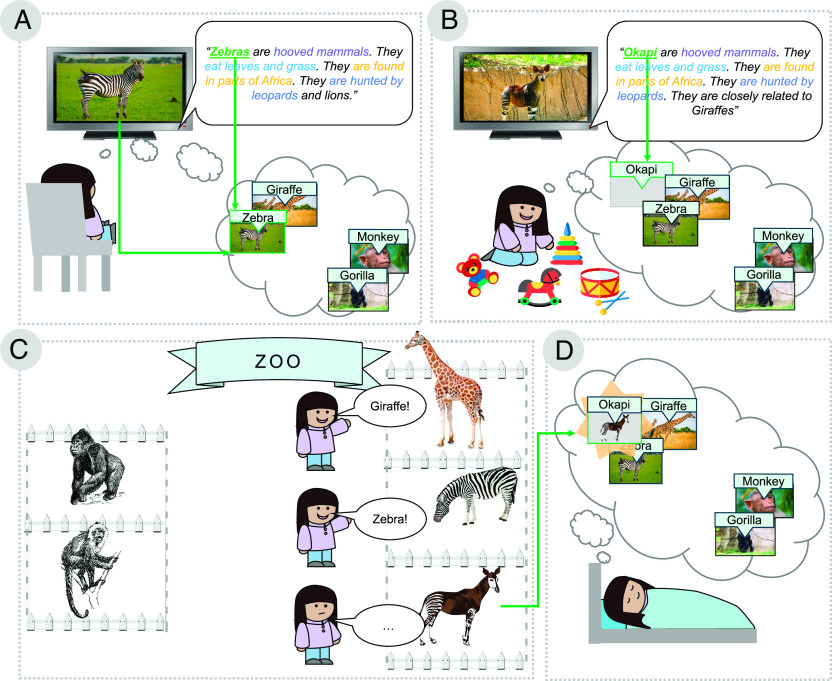
An illustration of how systems alignment could support asynchronous learning based on a child’s everyday experiences. Thought bubbles depict the child’s knowledge state, with visual and linguistic systems overlaid. (*A*) The child watches a nature documentary, where she learns about zebras from synchronous visual and linguistic input. Zebra is added to her knowledge state by this event-based learning process. (*B*) She begins playing with toys with her back to the television. While she is no longer watching the TV, she can still hear the documentary audio describing okapi. The descriptions of okapi and zebras are very similar, which leads to “Okapi” being positioned in linguistic space close to “Zebra.” Note that she does not have to understand the meaning of all words surrounding “Zebra” and “Okapi” for this similarity relation to be acquired. (*C*) Later, the child visits the zoo. From previous experiences, she can label the giraffe and the zebra. She sees an unknown animal in a nearby enclosure, which shares visual similarities with the giraffe and the zebra. (*D*) Using the asynchronous inputs in different modalities, she is able to infer that the unknown animal at the zoo is likely an “Okapi.” This is possible via alignment of visual and linguistic systems.

An example of this is shown in Fig. 2: based on a documentary voiceover heard on a prior day, a child at a zoo could use alignment to map a previously unseen animal to an animal name she has heard before. Alignment could also facilitate learning asynchronously via known processes of memory replay (27).

The intuition underlying systems alignment is that items that share similar visual contexts, such as a car and a truck, will also tend to share similar linguistic contexts. Because of the mirrored similarity relationships, it is possible to align two different systems (28, 29). The unique signature an item has in one system repeats in other systems. The consequences of an aligned system are substantial. For example, aligning visual and linguistic systems would allow one to infer the label for every visual object absent object-label pairings. In principle, simply having experience of each system, possibly separately, would be sufficient to infer the similarity relations within each system, which in turn would be sufficient to align the two systems.

We define a concept as a correct mapping across systems (e.g., the correct mapping between the word “car” and the corresponding visual object). We refer to the set of known concepts as the knowledge state. In practice, prior knowledge of some concepts, such as knowing the label “car” maps to an image of a car, facilitates or bootstraps systems alignment (Fig. 1). According to systems alignment, the more that is known, the easier it becomes to infer new knowledge. For example, based on knowing the mapping for car, and the unimodal similarity relationships between cars and trucks, we predict that a child could infer the name for a truck without ever experiencing the verbal label “truck” co-occurring with the visual experience of a truck (Fig. 1). Systems alignment may help explain why children’s vocabularies rapidly expand after around 50 words are known (30, 31).

One key question is whether the information present in our natural environment can support systems alignment for cross-modal learning. Early evidence demonstrated that there are redundancies in the information captured by linguistic and visual systems (32, 33). Roads and Love (28) have since answered this question in the affirmative, demonstrating that when systems—derived from the environment—were aligned, their mirrored similarity structure—which they referred to as an alignment score—was higher than for other (incorrect) mappings between the systems. Thus, in principle, an algorithm that maximized alignment score could achieve systems alignment. A second key question is whether people engage in systems alignment when learning. In a laboratory study using well-controlled materials, we recently established that adults, unprompted, do align systems in a learning task that could have been solved solely through event-based, supervised learning (34). This demonstrated that alignable systems are more readily learnable systems. Thus, here, we address a third question, namely, could children use systems alignment to help them learn the meaning of words?

Our consideration of systems alignment in a developmental context is different from prior work. Relevant prior work on fast-mapping demonstrates alignment effects on a local scale (e.g, “pass the chromium tray, not the blue one”, where “chromium” is a previously unknown label) (35, 36). Perceptual alignment has been explored as a signal in early adjective learning and has been found to aid learning in incidental learning contexts (11). Analogies between word forms may help children learn to read (37). But where prior work on analogy (38) and alignment processes (39) has been restricted to local contexts, we argue that systems alignment could be performed between entire systems of relationships, such as across modalities to promote cross-modal learning.

Besides alignment, prior work has identified a range of factors which influence how concepts are acquired. Constraints, such as the mutual exclusivity assumption, the taxonomic assumption, and the whole-object assumption (3, 4), are known to play a role in ambiguous labeling events. Lexical, phonological and semantic features—such as word frequency, phonological neighborhood size, and associations with other words—have all been found to be predictive of a concept’s age of acquisition (40–42). Structural analyses of semantic networks have also identified patterns in how conceptual knowledge develops in early life (43–45), but the influence of structural factors in unsupervised cross-modal learning has not yet been explored. Here, we consider whether systems alignment can explain aspects of how children acquire word meanings in a manner that complements existing explanations.

Throughout this contribution, systems are operationalised as GloVe embeddings (15) in both linguistic and visual modalities. GloVe embeddings are distributional semantic models, trained on co-occurrence data. GloVe’s performance on some language tasks corresponds closely with human performance (46). In our analyses, linguistic embeddings are derived from word co-occurrences in text corpora, and visual embeddings from object co-occurrences in visual scenes (28, 47). Further information on these embeddings can be found in *Materials and Methods*.

We take a systems alignment view, solely concerning ourselves with factors related to the structure of similarity relationships between items, within a system. If gage in systems alignment, then concepts that readily align across systems should be acquired more easily than those that do not (28), forming a basis for subsequent learning. We test the hypothesis that children’s early concepts are particularly well-suited for facilitating learning by systems alignment in our first simulation experiment using a forced-choice paradigm. To foreshadow our results, simulated agents whose early knowledge states comprise concepts that are acquired early by children better assimilate new conceptual knowledge through systems alignment. Furthermore, early acquired concepts are acquired via alignment with particular ease. These results were corroborated by simulations which used word embeddings derived from child-directed speech.

We proceed to investigate whether there are quantifiable structural underpinnings of this alignment effect within visual and linguistic systems. What is it about early acquired concepts and their relationships that allows for new conceptual knowledge to be more readily aligned? Our view predicts that knowledge states which yield distinctive similarity relationships for unknown concepts will be preferred in early life. In line with this prediction, structural analysis reveals distinctive characteristics of the similarity relationships of early acquired knowledge states. Finally, to assess the generalisability of these structural features for supporting alignment performance, we train generative agents to build knowledge states by optimizing these structural parameters. Consistent with our alignment-based view, we find that agents that build their knowledge states based on these structural features outperform all other agents in their ability to learn by alignment.

## Results

### Inference from Early Concepts.

We found that agents that built knowledge states based on children’s Age-of-Acquisition data (48) (AoA agent condition) performed better at inferring mappings between visual and linguistic systems using systems alignment than control agents.

**Fig. 3.**
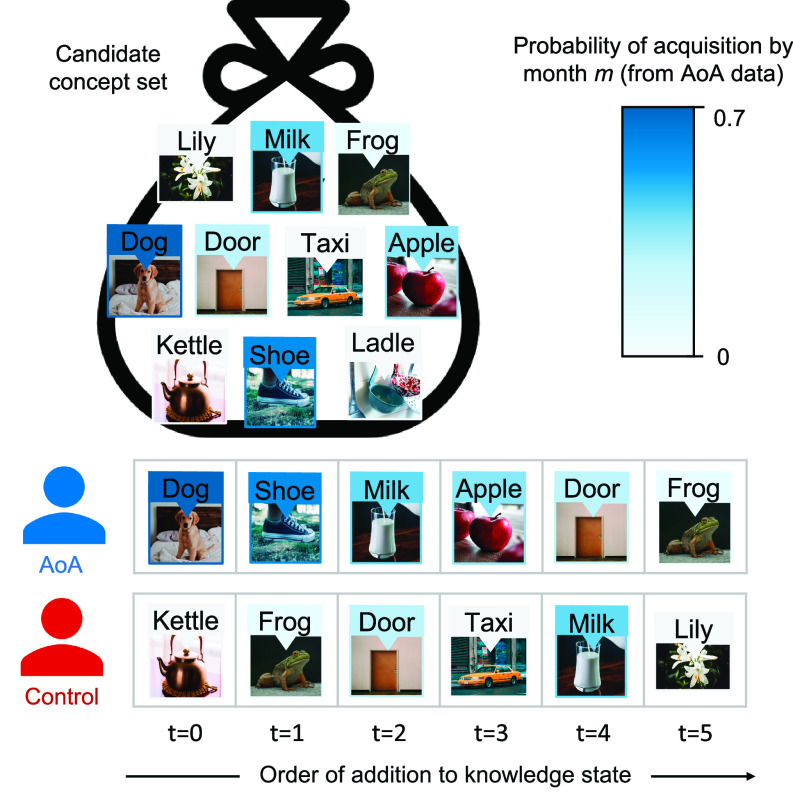
An illustrative example of how knowledge states expand in simulated agents. In this example, six concepts ($n_{m}=6$) are added to each agent’s knowledge state. The AoA agent’s knowledge state grows in accordance with the probabilities of each concept’s acquisition by month *m* in the AoA data (i.e., the agent acquires concepts typical of children). The Control agent ignores AoA information—the concepts added to its knowledge state are randomly drawn from the full set of concepts (*Materials and Methods*).

For each month of age *m* from 16 to 24, we simulated knowledge state development for agents by adding *n*_m_ concepts to their knowledge states, where *n*_m_ was the expected number of concepts acquired in that month from the Age-of-Acquisition data (*Materials and Methods*). AoA agents added concepts to their knowledge state in an order that mimicked human AoA data, by sampling concepts from distributions of concept acquisition probabilities. Control agents added randomly selected concepts to their knowledge states. Knowledge state simulation process is visualized in Fig. 3. Do children’s early concepts provide a strong foundation for inferring new concepts through systems alignment?

**Fig. 4.**
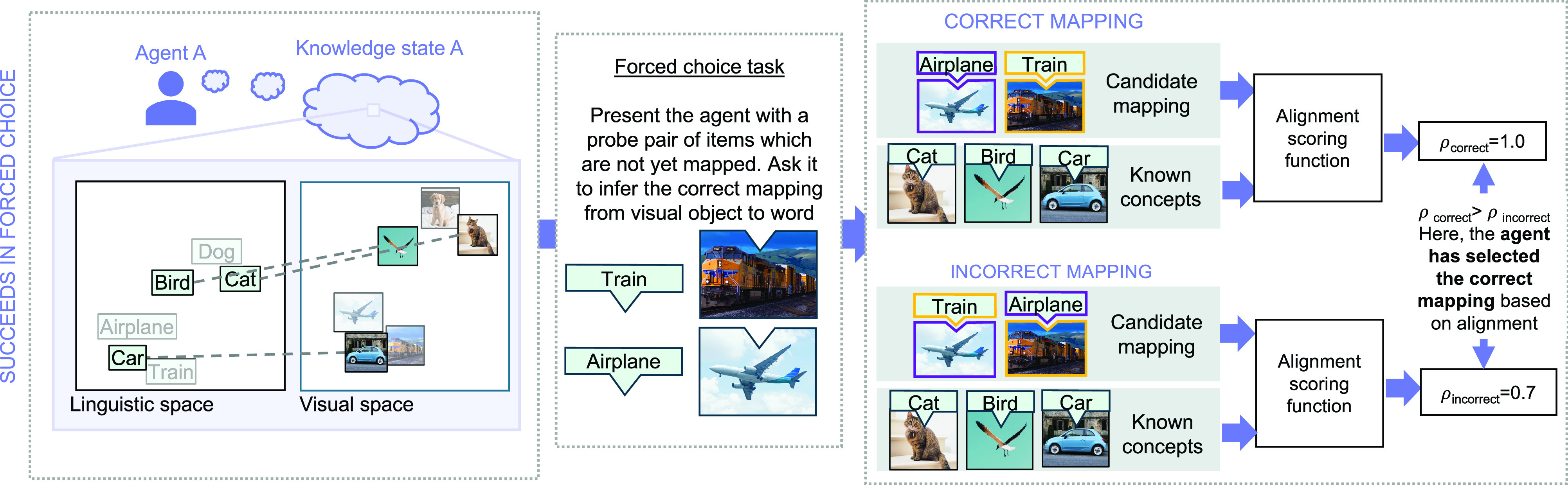
Example of the forced-choice task used to evaluate agents. In this example, Agent A’s knowledge state allows it to make the correct inference in the forced-choice task, using alignment. In the *Left* panel, the agent’s knowledge state prior to the forced-choice task is represented. Grayed-out images and words in the visual/linguistic spaces represent items which the agent has experienced separately in each modality, but which have not been mapped across systems. The next panel to the *Right* shows an example forced-choice task: In this case, agents are asked to infer which of two visual objects is an “Airplane” and which is a “Train.” The next panel shows how the agent attempts this inference. The agent obtains the alignment score for each candidate mapping of the probe items using the alignment scoring function. The alignment scoring function is discussed in detail in *Materials and Methods*. Agent A correctly identifies the appropriate mapping, because the alignment score for the correct mapping is higher than the score for the incorrect mapping. For an example of a knowledge state which would yield failure in this forced-choice task, see *SI Appendix*, Fig. S1.

Agents were evaluated each month in their ability to learn new concepts by alignment using a forced-choice paradigm. At each month, they were tasked with inferring the correct object–word mappings for novel probe pairs of words and visual objects (Fig. 4). They made each choice by examining the relationships between the probe items and the items in their current knowledge state, in each modality. They selected the object–word mapping where the pattern of relationships to existing items yielded the highest correspondence across modalities.

Probe concepts were sampled using two different probe conditions: either from the remaining AoA concepts (AoA probe condition) or from all remaining concepts (Unconstrained probe condition).

**Fig. 5.**
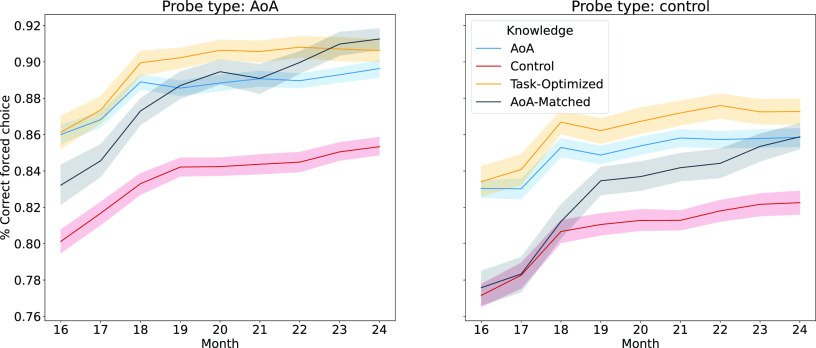
Results for forced-choice experiment for different agent types. Shaded areas represent 95% CIs across 100 agents for each agent type. AoA vs control: Blue lines represent performance for agents simulating AoA-based concept acquisition, and red lines represent results for control agents. Generative modeling: Orange and black lines represent results for structural agents. Black lines represent performance for the agents which are trained to match AoA acquisition statistics; orange lines represent performance for the agents which are trained to optimize probe pair performance.

The results are shown in blue (AoA) and red (Control) in Fig. 5. First, it’s striking that with only a handful of known concepts that both agents’ inferences are over 80% accurate in the forced-choice task (see *SI Appendix*, Table S1 for month-wise t-tests). This indicates that children, like our agents, could correctly label objects by aligning systems (e.g., visual and words), using similarity relationships to their known concepts. These results extend those of ref. 28 to suggest that systems alignment is useful for inferring unknown concepts and building useful priors or expectations.

Second, we found that the AoA agent was more effective than the control agent ($F\left(1,198\right)=347.48,P<.001,\eta_{p}^{2}=0.627$) and an advantage of AoA test probes ($F\left(1,198\right)=529.96,P<.001,\eta_{p}^{2}=0.719$). There was also a significant interaction between agent and probe type ($F\left(1,198\right)=11.83,P<0.001,\eta_{p}^{2}=0.069$) such that the AoA probe effect was heightened for the AoA agent. Complete ANOVA results are provided in *SI Appendix*, Table S2.

The same pattern of results was also observed when the word embeddings were replaced with embeddings derived from child-directed speech corpora (*SI Appendix*, Fig. S3).

### Structural Characteristics of Early Concepts.

Early AoA concepts better supported inference by systems alignment than a random sample. Here, we quantify which features distinguish AoA knowledge states from Control knowledge states in the hopes that the analysis illuminates the AoA advantage.

We considered a range of structural features for the knowledge states of both Control and AoA agent types. Features were derived from raw similarity relations of concepts and graphs of concepts’ close neighbors within the aforementioned embedding spaces. Graphs were constructed by retaining connections for concept pairs only where interconcept distance is below the 10th percentile of all interconcept distances. Where applicable, these features were calculated for each concept with respect to both a) the full system of all concepts, and b) the set of concepts already in the agent’s knowledge state at the point of acquisition. Additional features characterized the knowledge state as a whole, including its average coverage of embedding space dimensions and the distribution of node degrees within the knowledge state (see *SI Appendix*, Table S3 for the full table of features tested).

A logistic regression classifier was trained to predict if a knowledge state was sampled under the AoA or the Control condition. We used recursive feature elimination to identify the features which were most diagnostic in demarcating early acquired knowledge sets. Regression results are shown in Table 1.

**Table 1.** The β values of logistic regression after recursive feature elimination

Feature	β
Degree_knowledge_	7.21
Degree_full_	5.36
Mean(Dist_knowledge_)	−3.28
Mean dimension coverage	−7.43
Min(Dist_knowledge_)	−4.04
Min(Dist_full_)	−2.60
Skew(Degree_knowledge_)	4.76

The regression model was trained to classify sample knowledge states as early acquired (Y=1) or control (Y=0).

Recursive feature selection chose seven features: distance to closest neighbor in full system and knowledge state, mean degree in full system and knowledge state, degree distribution skew in knowledge state, mean distance in knowledge state, and mean dimension coverage of the knowledge state. The accuracy, recall, and precision of the resultant model were all over 96% for a balanced set of 900 Control and 900 AoA knowledge state samples, meaning that all models correctly classified a significant majority of samples on the basis of these features.

The regression analyses indicated that AoA concepts are distinguished by their dense neighborhoods. From a systems alignment perspective, density may be advantageous because it promotes stability in representations across initialisations. Embedding algorithms are sensitive to initial conditions such that the position of items within an embedding can vary across simulations. Human learners may also be affected by these and other factors, such as noise and the idiosyncratic nature of human experience. These initial conditions are likely to have greater impact on more distant interconcept relationships, as these relationships carry less meaning and are therefore likely to be more sensitive to noise. We confirmed the stability hypothesis: Concept pairs with greater interconcept distance also have greater variability in the interconcept distance across multiple embedding initialisations *SI Appendix*, Fig. S10), meaning that dense neighborhoods characteristic of AoA concepts are better suited to systems alignment. The stability of short-range interconcept relationships may explain why children acquire concepts with many semantic neighbors in early life (43, 44).

### Learning with Generative Agents.

Having identified structural features that distinguish early acquired concepts, we explored whether these features could be used by agents to build knowledge states that support inference by systems alignment. Whereas the previously considered AoA agent sampled concepts in accord with those children acquire, these new structural agents are not tied to the particular concepts children learn but are instead sensitive to their underlying structural features. Should these structural agents perform well, it would support the notion that properties of embedding spaces, including structural factors, influence which concepts are most readily acquired by systems alignment.

We considered two structural agents, the AoA-Matched agent and Task-Optimized agent. Both structural agents optimized a set of target values for the structural features identified in the previous section ($\hat{x}$), and a set of feature weights (**w**). $\hat{x}$ and **w** are henceforth referred to as the internal model. A generative distribution across candidate concepts was obtained by calculating each candidate concept’s weighted distance from the target feature values $\hat{x}$. This distribution determined the probability of each concept’s selection for the knowledge base. The agent types differed in the loss function that was used to optimize the internal model: The AoA-Matched agent was trained to generate knowledge states which mimicked children’s early concepts (i.e, maximized similarity between the generative distribution and probabilities of concept acquisition from the AoA data); the Task-Optimized agent was trained to maximize performance of the resultant knowledge states on the forced-choice task. Further details are available in *Materials and Methods*. The Task-Optimized agent serves as an upper bound on the forced-choice performance which can be achieved by optimizing the internal model, to which the AoA-Matched agent’s performance can be compared.

After each agent model was trained, their internal model was frozen. They then generated concept acquisition trajectories by sampling from the generative distributions calculated from the optimized internal models (*Materials and Methods*). As the agent sequentially selected concepts, it was tested each month using forced-choice tasks composed of AoA and Unconstrained probes.

Forced-choice results for the generative paradigm are shown in Fig. 5. Significance tests for all agent comparisons are provided in *SI Appendix*, Table S4. As expected, the Task-Optimized agent outperformed the AoA-Matched agent. Indeed, the Task-Optimized agent also outperformed the previously considered AoA and Control agents. The success of a structural agent is consistent with the notion that structural factors relevant to systems alignment play a role in determining children’s early concepts. The AoA-Matched agent’s performance was comparable to AoA agent in the AoA probe condition, whereas its performance only converged later in learning in the unconstrained probe condition. One explanation is that the structural features for the AoA-Matched agent were optimized to discriminate early acquired concepts from other concepts, which may have discarded features that would be useful under more general testing conditions (i.e., when tested with probes from the unconstrained set). The Task-Optimized agent overcame this issue by tuning features diagnostic of AoA concepts. The structural features of concepts that children acquire have also been shown to vary with the time of acquisition (44), while our agents’ internal models are static. This may explain the delayed convergence of the AoA-Matched and empirical AoA agent performance.

### Concepts Chosen by the Agents.

The structural agents did not rely solely on concepts in the AoA set with 57% and 62% of concepts being early acquired in the final knowledge states of Task-Optimized and AoA-Matched agents respectively (*SI Appendix*, Fig. S6). AoA trajectories are not the only learning path which yields success using these structural features—the agent results demonstrate that the solution space is larger than what is observed empirically with children as modeled by the AoA agent. Despite not being fully reliant on AoA concepts, both the AoA-Matched and Task-Optimized agents did select AoA concepts significantly more often than the 33% rate of the control agent (t(198)=48.9,P≪0.01 and t(198)=43.7,P≪0.01, respectively). This result from the Task-Optimized agents, which were not trained to select AoA concepts, provides support for the conclusion that children learn concepts whose structural features facilitate learning via systems alignment. The proportion of AoA concepts selected by the structural agents declined over learning (*SI Appendix*, Fig. S7).

The two structural agents had different priorities when selecting concepts for acquisition: The Task-Optimized agent, which performed better in forced-choice, prioritized learning concepts which had many close neighbors in the full system; the AoA-Matched agent, on the other hand, prioritized acquiring concepts which had low mean distances from other concepts in the existing knowledge state and many close neighbors within the knowledge state (*SI Appendix*, Fig. S8). This suggests that Task-Optimized agents achieved their superior performance by focusing on concepts which have dense similarity neighborhoods. AoA-Matched agents were prone to select knowledge states with low coverage, as was shown to demarcate AoA concepts in the regression results, but this was not true of Task-Optimized agents, indicating that while low coverage may be a feature of early acquired knowledge states, it does not necessarily contribute to the enhanced alignment effect.

We examined the semantic category coverage of the concepts learned by each agent type (details available in *SI Appendix*). The AoA-Matched agents show a similar distribution across categories to the AoA agents, while the Task-Optimized agents have a tendency to focus on fewer semantic categories, as indicated by low cross-category entropy (*SI Appendix*, Fig. S9). This implies that the Task-Optimized agents prefer specialism/depth of knowledge within categories, as opposed to covering all category bases as a priority for knowledge acquisition. Taken together with the finding that densely connected concepts appear to be key for successful alignment, this suggests that AoA concepts may have to strike a balance between features which promote alignment and features which are crucial for knowledge states to address the demands of real life (e.g., having conceptual understanding across categories).

## Discussion

We demonstrated that aligning systems can aid the acquisition of conceptual knowledge. By matching interconcept relationships alone, it is possible to infer the meanings of concepts with over 80% accuracy in a forced-choice task with a knowledge state containing only 21 known concepts. This startling result, along with prior work on systems alignment (28, 34) and supportive brain imaging findings (49), suggests a revised account of human learning. Rather than relying on event-based learning, humans could capitalize on signals from the alignment of conceptual systems, which would enable asynchronous events to be linked in an offline manner, akin to how neuroscientists characterize memory replay (27).

We found that children’s early acquired concepts provided knowledge states that better supported alignment than randomly sampled knowledge states (i.e., AoA vs. Control agent results, Fig. 5). In turn, early acquired concepts were easier to learn by alignment than later-acquired concepts (i.e., AoA vs. Unconstrained probe type results, Fig. 5). These findings suggest that children could engage in the types of unsupervised learning involved in systems alignment, which would lead to a preference for concepts forming alignable systems. A complementary possibility is that children are biased to acquire alignable systems of concepts based on some structural property of these knowledge states.

In accord with this second possibility, we found that children’s early (AoA) concepts were distinguished from other concepts by certain structural features, such as being densely packed and interconnected (Table 2). We predicted that these features were particularly beneficial for systems alignment. To evaluate this possibility, we built agents that used these features to select concepts to learn through systems alignment (Fig. 4). As predicted, these agents were more effective than agents that randomly sampled concepts (Fig. 5). The AoA agent patterned after children’s acquired concepts performed nearly as well as the Task-Optimized agent which indicates that children’s early concepts provide a knowledge base highly suited to (and perhaps shaped by) systems alignment.

**Table 2.** Number of concepts acquired in each month, based on the mean number of concepts known in each month of the WordBank dataset

Month (*m*)	16	17	18	19	20	21	22	23	24
Concepts acquired in month *m* (*n*_*m*_)	17	3	15	9	4	6	14	9	6
Cumulative concepts (*N*_*m*_)	17	21	36	45	49	55	68	77	83

Values are rounded to the nearest whole number of concepts.

These structural agents were constrained to follow the feature patterns characteristic of children’s early concepts rather than learn the specific concepts children do. Their success demonstrates that there are multiple paths to successful learning by alignment. Indeed, the specific concepts learned by the structural agents differed from those children learned (*SI Appendix*, Fig. S6). When we use our structure-based models to generate sequences using only concepts which are not in the AoA dataset at all, they still achieve forced-choice performance with up to 90% accuracy at the maximum knowledge state size tested (*SI Appendix*, Fig. S11). In summary, while children’s early concepts form a readily aligned system, there are many other knowledge states that also support systems alignment.

Unlike the artificial agents, children likely face a trade-off between the concepts which are easiest to integrate with their current knowledge by alignment, i.e., those with dense connections- and those which they must learn in order to gain a functional understanding of their world. We found evidence of this trade-off by comparing the structural preferences of agents which were trained to mimic early concept acquisition and agents which were trained solely to optimize alignment performance. Agents which mimicked real-world concept acquisition struck a balance between densely connected knowledge states and knowledge states which spanned semantic categories, while agents optimizing alignment performance honed in on a narrow range of semantic categories, favoring connection density.

We focused exclusively on systems alignment, which we view as an exciting and under-explored avenue for learning. We fully acknowledged that other factors shape early concept acquisition. Supervised episodes, and other aforementioned event-based inputs, undeniably affect children’s learning. Rather than be in opposition, systems alignment is compatible with other forms of learning, including event-based learning. For example, Aho et al. (34) demonstrated that alignment signals facilitate learning even when they are not necessary for success, and are accompanied by event-based signals. Children’s learning likely reflects a mix of systems alignment and event-based learning. In our own results, we found that AoA concepts tend to be higher frequency, which we take as a marker of event-based learning. Thus, children’s early concepts show indications of both systems alignment and event-based learning. When we limit our simulations to non-AoA concepts that tend to be lower frequency systems alignment continues to perform well (*SI Appendix*, Figs. S4 and S5), suggesting that it may be possible to disentangle these forms of learning that are likely intertwined in natural environments.

Systems alignment can explain how learning is possible from weak supervisory signals. In naturalistic environments, weak signals may come in the form of ambiguous (2, 13) or infrequent (25, 50) labeling events, or indeed from the context-specificity of early language (26, 51). These signals could constrain systems alignment processes by suggesting links between systems and restricting the set of candidate solutions. In turn, systems alignment could help constrain weakly supervised mapping problems by favoring mappings that mirror similarity relationships across systems.

Previous work has found that words that appear in diverse contexts and are densely connected are more likely to be acquired (52). Such results naturally follow from systems alignment. We found that relationships in embeddings across multiple initialisations are most stable for shorter-range relationships, which would make knowledge bases consisting of densely packed concepts most reliable for systems alignment. This inherent sensitivity to initial conditions and noise in learning systems may privilege densely packed concepts as found in children’s early concepts. We hope systems alignment can offer a valuable perspective on other empirical findings.

Learning via systems alignment remains to be tested in children under controlled conditions. Our results invite directed laboratory studies to evaluate whether children’s learning is accelerated by systems alignment. Like our agents, we predict children should be able to infer novel mappings between objects and labels using systems alignment.

We made the simplifying assumption that each embedding or similarity space is constant over time. While there is evidence that similarity spaces apply over development – co-occurrence statistics derived from child-directed speech generate adult-like word embeddings (21, 22) – and our own analysis showed that the alignment benefit for early acquired concepts is also observed when using child-directed speech embeddings (*SI Appendix*, *Text*), one would expect some changes in these spaces over learning. Infant environments, while certainly correlated with adult environments for the concepts they are exposed to, are more constrained than adult environments, and semantic spaces will develop over time.

A limitation of this work remains in the fact that the visual object embeddings are not specific to children’s environments. Although the similarity relationships between the concepts studied here are likely to be largely re-capitulated in such embeddings, these embeddings may align even better with child-directed speech embeddings. Future work could infer more child-like visual representations to explore how alignment signals manifest in early similarity space.

Additionally, while our visual embeddings are based on visual object co-occurrences, the semantics of the visual space may instead be captured by embedding other kinds of visual information, such as how objects co-occur with actions and contexts (26, 51) or objects’ perceptual features (32). Embeddings based on these types of visual information may better capture how children judge visual similarity, which may improve systems alignment to linguistic spaces (33).

To simplify, we considered conceptual understanding as “all-or-nothing”: When a word-image mapping is known, the concept is “understood.” Instead, conceptual understanding, and indeed cross-modal mappings themselves, are likely graded. One possibility is that systems alignment may provide informative priors for concept learning, thus facilitating event-based learning as demonstrated in ref. 34. For example, possible alignments that lead to higher alignment scores could be assigned higher priors. In turn, event-based learning constrains systems alignment by expanding the knowledge base. These principles could benefit machine learning systems using alignment-informed priors for multimodal learning.

## Materials and Methods

### Materials.

#### Word embeddings.

As systems alignment is driven by similarity relationships between concepts, not knowledge of the concepts themselves. A child may have different knowledge of a car, a truck, and an alligator than an adult (53), but, like an adult, the child will judge the car as more similar to the truck than the alligator. Preserving similarity relationships is all that is required for systems alignment. With this in mind, we compared large-scale pretrained word embeddings to word embeddings derived from child-directed speech, to choose the most suitable for this study.

##### Pretrained word embeddings.

The pretrained word embeddings were 50-dimensional GloVe text embeddings (15). These embeddings are trained on 6 billion tokens from the Wikipedia2014 + GigaWord5 text corpus. The resultant vocabulary size is 400,000 tokens. These embeddings were selected as the primary text embeddings for the analyses in this paper, owing to a) the large size of the training corpus, which has been shown to significantly impact embedding stability (54), b) their established correspondence to human semantic judgments of language (46), and c) the finding that child-directed speech embeddings correlate as highly with these embeddings as these embeddings do with themselves (*SI Appendix*, Fig. S5).

##### Word embeddings from child-directed speech.

It is important to consider the use of word embeddings from child-directed speech as possible relevant model of linguistic space for this study.

We inferred embeddings from the North American English subset of the CHILDES database (55). Each transcript in the database was treated as a document. After preprocessing to extract child-directed speech inputs and remove punctuation, the compiled corpus was inputted into the GloVe algorithm. The resultant corpus contained 4 million tokens, and had a vocabulary size of 12,252. The algorithm was run with an output vector size of 50 and a window size of 10. The algorithm ran for 1,000 iterations. The minimum count of word occurrences in order for a word to be included in the GloVe algorithm was 5.

Child-directed speech embeddings were less suited for the main analyses presented in the article text. This was because a) an analysis of the embeddings demonstrated that the corpus size was not sufficient to guarantee the stability of interconcept relationships (54) (see *SI Appendix*, *Text* for the analyses), b) it is unclear whether embeddings derived from child-directed speech capture children’s knowledge. Given this, and the finding that child-directed speech embeddings correlate as highly with the embeddings from the general corpora as those general embeddings do with themselves (*SI Appendix*, Fig. S5), the general pretrained word embeddings were used.

Despite this, we did conduct a forced-choice experiment with these embeddings, to corroborate the result that early acquired concepts were superior in their ability to facilitate alignment, compared to randomly selected concepts (see *SI Appendix* for full results).

#### Image embeddings.

The image embeddings used here are those used in ref. 28, derived by applying the GloVe algorithm (15) to the Open Images V4 dataset (boxes subset) (47). Open Images V4 is comprised of approximately 9.2 million images, all annotated to identify which of over 19,000 object classes they contain. (28) construct a co-occurrence matrix by counting the images in which each object class co-occurs with each other class. This matrix is inputted to the GloVe algorithm, which generates the 10-dimensional image embeddings we use.

#### Age-of-acquisition data.

Age-of-acquisition (AoA) data taken from ref. 48’s WordBank dataset. WordBank aggregates experimental results using MacArthur-Bates Communicative Development Inventories (MB-CDI) (56). We used the English (American) dataset, containing data from linguistic development trajectories of 8,300 children. Specifically, we used the item trajectory dataset, which reported the proportion of children who could produce each word by each month of age. These data are obtained from parental reports of children’s word production. This dataset contains monthwise probabilities of the acquisition of 680 words overall. We preprocess the dataset by taking the subset of WordBank words which exist in the intersection of our word and image embeddings. There are 418 words in the word/image intersection, and of these, 138 words are present in the WordBank dataset. These 138 words comprise the AoA concept set, and the full set of 418 concepts in the image/word embedding intersection intersection comprise the control concept set.

AoA data are available for children from 16 to 30 mo of age. However, as the MB-CDI is an index of representative words for early vocabulary, and not a comprehensive review of a child’s entire vocabulary, it is known that MB-CDI results diverge from true vocabulary size as MB-CDI scores increase (and, typically, as a child gets older) (56, 57). This is because as the vocabulary expands, the representative words which comprise the MB-CDI become less likely to capture the idiosyncrasies of an individual child’s vocabulary. Mayor & Plunkett modeled the extent of the divergence (58), and provided estimates for the proportion of the vocabulary which is not captured within the MB-CDI at each month of age.

For our probabilistic interpretation of the MB-CDI data, we require the assumption that the probability of a child having acquired any concept outside of the index is approximately 0. Therefore, all of our modeling and analyses are performed using WordBank data for months 16 to 24 only, where the index is likely to capture close to 100% of a child’s vocabulary.

We assume that the order in which words are produced corresponds to the order in which words are “known.” For our purposes, a word is “known” when a correspondence is established to its visual form from its linguistic form (i.e, an agent can correctly label a picture of a dog as a “dog”). Estimating children’s knowledge bases using production norms likely introduces noise into our analyses and may underestimate semantic knowledge because other factors, including phonological, will influence which words children produce.

### Methods.

#### Alignment score.

**Fig. 6.**
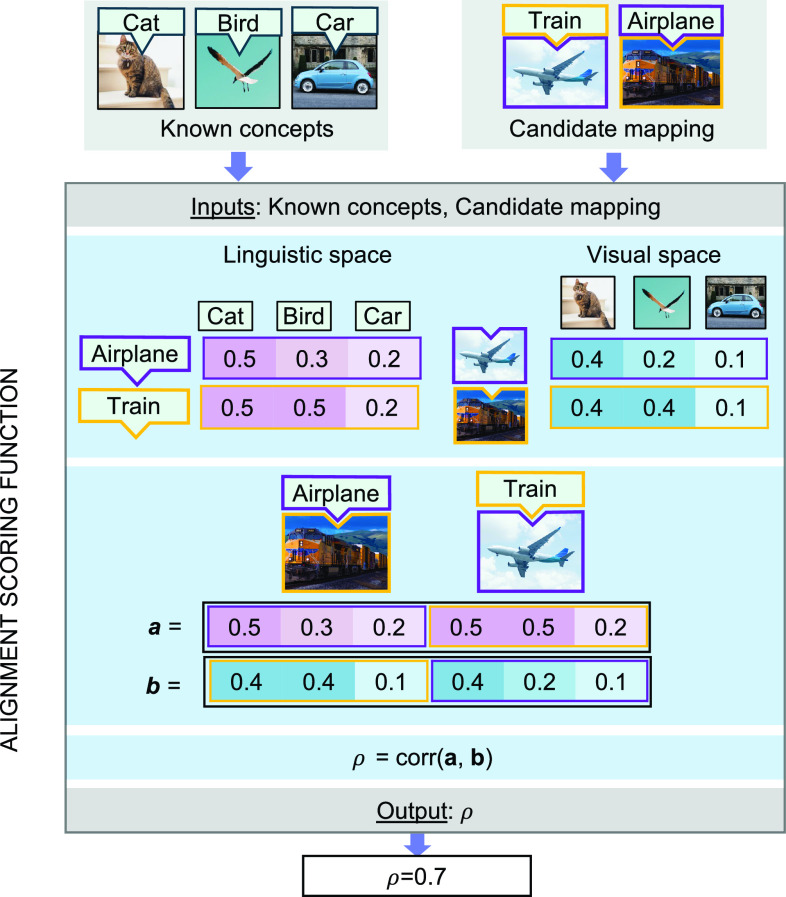
Interconcept distance correlation across modalities, for a candidate forced-choice mapping (in this case, an incorrect mapping), using an agent’s knowledge state. The higher the correlation, the higher the alignment score for the candidate mapping. For this example, the relevant systems are visualized in Fig. 4. First, the agent retrieves the interconcept distances for the probe items with respect to its known items in each modality. Then, the similarity relationships in each modality are concatenated in the order determined by the candidate mapping. The resultant vectors are correlated across modalities. The chosen mapping is the one which maximizes the correlation between similarity vectors across modalities.

In the general case, the alignment score between two systems is the Spearman correlation, $\rho_{s}$, between the upper triangular portions of the pairwise distance matrices within each system, where the order of items in the pairwise distance matrices is based on the known mappings between the systems (in this case, the items in image and word embedding spaces). For the forced-choice experiment, the positions of all but the two probe items in each system are fixed. Therefore, the candidate mapping that would yield the higher alignment score can be determined by the Spearman correlation of the concatenated matrix columns corresponding to the probe items across systems. The order of the concatenation is determined by the candidate mapping. This is visualized in Fig. 6.

#### Forced-choice experiment.

To simulate knowledge trajectories, we calculated the mean number of concepts $n_{m}$ acquired in each month *m* of the Age-of-Acquisition data. This is achieved by summing the probabilities of acquisition across all concepts in each month, $n_{m}=\sum_{i=0}^{N}p_{i,m}$, where N is the total number of concepts in our AoA set, and rounding to the nearest integer. This produces the sequence given in Table 2. At each month for each agent type, we therefore have a simulated knowledge state which contains $N_{m}$ concepts. We generate sequences of acquired concepts under two conditions:

- AoA: New items are selected from a probability distribution across items in the WordBank dataset (48). WordBank aggregates word-production results reported on the MacArthur-Bates Communication Development Inventory (MB-CDI) across numerous studies, for infants from 16 to 30 mo of age. The probability distribution is generated by normalizing the probabilities of acquisition for all concepts in the WordBank inventory which have not yet been added to the simulated sequence, such that the probabilities sum to 1.
- Control: New items are randomly selected from all items in the intersection of word and image embeddings which are not present in the WordBank dataset (i.e., all concepts which are not early acquired), and which have not yet been added to the simulated sequence.

Probe pairs are also generated under two sampling conditions:

- AoA: Probe items are selected randomly from the set of concepts in the WordBank dataset which do not exist in the knowledge state.
- Unconstrained: Probe items are randomly selected from all items in the intersection of word and image embeddings which do not exist in the knowledge state.

Both probe conditions are tested on both agent conditions, with 100 simulated agents for each condition. Month *m* and probe condition are within-subjects factors; agent condition is a between-subjects factor. This yields a two-way repeated measures design.

#### Structural analysis of knowledge states.

A full table of the features tested in this analysis is provided in *SI Appendix*, Table S3. Most features are averages of concept-wise features taken across the concepts in the knowledge state. These fall into one of two broad categories:

- Global similarity features: These are features based on similarity relationships in the full system of concepts. These features are rooted in the similarities between each concept and others in the system. For example, the mean global distance for a concept i would be the mean of i’s distance to every other concept in the system.
- Neighborhood graph features: These are derived from graphs constructed from only the shortest-range interconcept relationships (or, in other words, concepts’ immediate neighborhoods). We build a graph G, whose nodes are concepts within an embedding space, by retaining the vertices for the 10% of smallest interconcept distances, based on the similarity matrix.

Note that the graphs we generate in this study are not necessarily connected; therefore, some graph measures such as smallworldness are not applicable in our case. Clustering and betweenness measures were obtained using networkx in Python (59). While we explored clustering and betweenness results for the AoA vs control knowledge states, we excluded these variables from selection by the logistic regression model due to the computational demands of calculating them for model training. This exclusion had no impact on the performance of the model selected.

All features were normalized to fall in [0, 1]. Logistic regression was performed using scikit-learn in Python (60). We took an 80/20 training/test split of knowledge states. Logistic regression was performed with a liblinear solver and L2 loss, where the maximum number of iterations was set to 10,000. When applying recursive feature selection, values of k (number of features included in the model) from 1 to the full feature set were tested, and the and the value which minimized each model’s Akaike Information criterion (AIC) was selected. The value k=7 was selected. The model achieved classification accuracy of 0.98%, recall of 100%, precision of 97% f1 of 0.98% for a balanced set of samples.

#### Generative modeling.

Both structural agents were set up to learn a vector of target values $\hat{x}\in R^{k}$ for the structural features we identified as being predictive of early acquisition, where k is the number of features available to learn (k=7). We also learned a weight vector $w\in R^{k}$, which captured the relative importance of each feature. The agent training process is visualized in *SI Appendix*, Fig. S2.

The key distinction between the AoA-Matched and Task-Optimized agents was the loss function that is backpropagated to optimize the agent’s internal parameters. The AoA-Matched loss term pressured the agent’s probability of acquiring each concept to match the real probability of acquisition from the AoA data. Meanwhile the Task-Optimized agent aimed to directly optimize performance on the forced-choice task by using a loss term which pressurized the model to maximize the margin between alignment scores for correct and incorrect mappings ($\rho_{\text{s, correct}}-\rho_{\text{s, incorrect}}$) across a randomly selected set of forced-choice problems. We call this loss term the soft alignment loss.

##### AoA-Matched agent.

The loss for this model is the total MSE between a) the model’s estimated probabilities of each concept being included in the knowledge state by the end of month *m* and b) a set of bootstrapped probability distributions sampled from the WordBank acquisition probabilities (see below). The model’s estimate of the probability of an item already selected for the knowledge state being in the knowledge state by the end of month *m* is set to 1, and the probability distribution across remaining candidate concepts which is outputted by the current model determines the remaining probabilities. We train R=5 model restarts. Models are trained for 150 epochs. An Adam optimiser with a learning rate of 0.003 was used for training. We select the best model based on validation loss averaged across the final 5 epochs.

For comparison to the compare the model’s outputted probability distributions, we take bootstrap samples from the probability distributions of concept acquisition in WordBank. This is necessary so that we have training and validation data for model selection across restarts. It also builds in an acknowledgment of the fact that the WordBank dataset is itself a single sample from the underlying population distribution of word acquisition probabilities. The bootstrapping process is as follows:

- We generate B=1000 bootstrapped probability distributions, ${\tilde{P}}_{b}\left(X\right)$.
- For each one, we sample 300 AoA sequences using the procedure described above and visualized in Fig. 4. Then we calculate ${\tilde{P}}_{b}\left(X\right)$ from these generated sequences, by calculating the proportion of sequences in which each concept was acquired by each month.
- Generate train and validation sets of bootstrapped distributions (70/30 split for train/test).

As the magnitude of the loss varied by month, we aimed to normalize the loss month-wise such that no month was disproportionately favored in optimisation. To achieve this, we sampled 5,000 MSEs for each month *m*, by randomly selecting $n_{m}$ concepts and calculating the average MSE between the resultant probability vector and bootstrapped probability distributions. We then calculated a z-score for the loss term using the mean, μ, and SD, σ, of MSEs acquired for the relevant month. To ensure that no loss was below zero, we subtracted the z-score of the theoretical minimum MSE (0) from all MSE z-scores. This constituted the final loss term for the AoA-Matched agent.

##### Task-Optimized agent.

To train the optimal model, we backpropagate a soft alignment loss across a sample of probe pairs, where the alignment loss is the extent to which the incorrect alignment score is greater than the correct alignment score, averaged across pairs. The larger the margin for the correct alignment score (i.e., the clearer the correct answer is), the smaller the loss becomes. The alignment loss is soft because it is weighted by the candidate concepts’ probabilities of being selected for the knowledge state:

- For the optimal agent, the loss requires a sample of test pairs for the forced-choice experiment. Therefore, on each backpropagation step, we segment the remaining concepts into the “candidate set,” the “train set” and the “validation set.” The candidate set contains 300 concepts, and is comprised of the $n_{t}$ concepts in the current simulated knowledge state, and $300-n_{t}$ concepts randomly selected from the remaining concepts.
- This leaves 59 concepts for each of the training and validation concept sets, from each of which 750 random pairs of concepts are then sampled to serve as the testing and validation slates respectively.
- As in the training procedure for the structural agent, at each timestep we obtain a vector across all candidate concepts, whose value represents each concept’s probability of being in the knowledge state at t+1. Therefore, for concepts which have already been acquired, the value of this vector is 1; for any concepts which have not yet been acquired, the value is determined by the probability obtained from the generative score.
- This is the same probability vector used in the structural agent’s training process. In the case of the optimal agent, this probability distribution is used to weight the contributions of interconcept distances to the alignment score.
- We obtain the pairwise probability matrix $p^{T}p$, and use this to weight the Spearman correlation in the alignment score calculation for the two permutations of the test pair mapping.
- At each timestep, we backpropagate this soft alignment loss to update the target variable vector x and the weight vector w.

As before, we train R=5 model restarts to minimize this alignment loss, and models are trained for 150 epochs. An Adam optimiser with a learning rate of 0.01 was used for training. We select the best model based on validation loss averaged across the final 5 epochs, where validation loss is soft alignment loss calculated on a validation set of forced-choice items.

Following the observation that the magnitude of the soft alignment loss increases with the number of concepts in the knowledge state, we normalized the alignment loss terms within each month. To achieve this, we took samples of alignment loss values for each month as follows:

- For each of 5,000 samples for each timestep t, we sampled a pseudo-partition of 300 concepts, from which we sampled a knowledge state of size $n_{t}-1$.
- We then gave all of the selected concepts a probability of 1, and generated a uniform distribution across the remaining concepts in the pseudo-partition, just as is done prior to the soft alignment score calculation in training.
- We then calculated the difference between the correct and incorrect alignment scores for a randomly selected pair of concepts from outside of the partition set for each sample ($s_{\text{incorrect}}-s_{\text{correct}}$).

To normalize the loss terms in model training using the distribution of these differences, we calculated a z-score for the ($s_{\text{incorrect}}-s_{\text{correct}}$) component of the loss term, using the μ and σ parameters from the sampled distributions for the appropriate month. To ensure that no losses were below zero, we added the z-score of the theoretical minimum value of this component of the loss term which is −2 ($\text{min}=\text{min}\left(s_{\text{incorrect}}\right)-\text{max}\left(s_{\text{correct}}\right)=-1-1=-2$), to the difference in each month. This yielded the loss term that we optimized for the Task-Optimized agent.

##### Model training.

For each month *m* in the AoA data, we sample new concepts one at a time from probability distributions based on their proximity in structural feature space to the current target values $\hat{x}$, weighted according to the current estimate of feature importances. When selecting a new concept to add to the knowledge state according to our current model, we construct a generative score vector for the candidate concepts $s\in R^{n_{c}}$ (where $n_{c}$ is the number of candidate concepts for acquisition) as follows:

- Obtain feature matrix $A\in R^{n_{c}\times k}$ for the candidate knowledge states which result from adding each of the $n_{c}$ concepts in candidate concept set (i.e, all concepts which have not yet been acquired) to the current knowledge state.
- Obtain distance matrix $D=A-\hat{x}J_{1,n_{c}}$, where $\hat{x}\in R^{k}$ is the model’s current best estimate of target feature values and $J_{1,n_{c}}$ is an $1\times n_{c}$ matrix of ones. $D\in R^{n_{c}\times k}$, and captures the distance of the candidate knowledge state from the target value in each dimension.
- Get generative scores for all concepts, s=−Dw, where w is the current estimate of feature weights. The generative score calculates the weighted similarity between sampled items’ feature values and the current target values for each feature.
- Generate probability distribution across candidate concepts by taking the softmax of normalized scores (softmax temperature parameter $T=5\times 10^{-2}$).
- Sample a concept from this probability distribution and add to knowledge state.

At the end of each month, where the number of acquired concepts in simulation matches the average number of concepts acquired from the WordBank dataset, we backpropagate our loss.

##### Sequence generation.

Once a model has been trained, concepts are selected by sampling from the generative score distribution for the final target values for structural features, using the same process described in Model Training above.

## Supplementary Material

Appendix 01 (PDF)

## Data Availability

Simulation data; embedding data have been deposited in Alignment_in_AoA (https://doi.org/10.17605/OSF.IO/32JTV) (61).
