# Supplementary material for: Signatures of cross-modal alignment in children’s early concepts
Source: Proc Natl Acad Sci U S A. 2023 Oct 11;120(42):e2309688120. doi: 10.1073/pnas.2309688120 (PMC10589699; doi:10.1073/pnas.2309688120)
Supplement: Supplementary file 1 — Appendix 01 (PDF) [file pnas.2309688120.sapp.pdf]

Supporting Information for

A role for cross-modal alignment in early concept learning

Kaarina Aho, Brett D. Roads and Bradley C. Love

Kaarina Aho.
E-mail: kaarina.aho.18@ucl.ac.uk

This PDF file includes:

Supporting text
Figs. S1 to S14
Tables S1 to S9
SI References

Supporting Information Text

This supporting information contains analyses which expand upon and support the main article text. This includes a replication of the result of our first analysis - that children's early concepts are more conducive to learning by alignment than other concepts - using child-directed speech embeddings, and analysis of the suitability of these child-directed speech embeddings for the rest of the contribution. It also contains in-depth analyses of the concepts learned by our structural agents. Details of statistical analyses presented in the main text are provided in the tables.

Results using child-directed speech. To explore the potential effect of the size of the child-directed speech corpus obtained from CHILDES (1) on the resultant embeddings (described in *Materials and Methods*), we conducted an analysis using a subset of the training corpus for the pre-trained GloVe embeddings. The key finding was that CHILDES embeddings correlated as well with the pre-trained GloVe embeddings as the GloVe embeddings did with themseleves, when the training corpora were of comparable size. The embeddings trained on small corpora were found to have unstable similarity relationships compared to the large-scale pre-trained GloVe embeddings.

Sampled enwik8 embeddings for comparison. For the purposes of comparison, we ran parallel analyses using the enwik8[*] dataset. The enwik8 corpus consists of a Wikipedia dump, which is also found in the training data for original pre-trained GloVe embeddings. For our purposes, enwik8 was pre-processed such that each Wikipedia article was represented as a distinct document.

We randomly sampled 20 CHILDES-sized sample corpora from the enwik8 dataset, and inferred an embedding for each using the GloVe algorithm, using same GloVe parameters as were used for the CHILDES embeddings.

Forced-choice experiment. The results for the forced-choice experiment conducted using the resultant child-directed speech embeddings are shown in Figure S3, with ANOVA results provided in Table S4. The results of the experiment echo the results when the pre-trained GloVe embeddings were used: we find a significant main effect of agent type ($F(1, 198) = 1165.70, p < .001, \eta_p^2 = 0.850$) and probe type ($F(1, 198) = 12.03, p < .001, \eta_p^2 = 0.070$), as well as a significant agent $\times$ probe type interaction ($F(1, 198) = 6.90, p = .009, \eta_p^2 = 0.049$).

Embeddings derived from a sample corpus drawn from enwik8, of comparable size to the CHILDES corpus, did not yield the same results. The results for this corpus are shown in Figure S4, and ANOVA results are provided in Table S5.

Comparison to original GloVe embeddings: noise ceiling analysis. Prior work shows that corpus size can have a substantial impact on the reliability of inter-concept relationships (2). To meaningfully evaluate the similarity of the child-directed speech embeddings and the original GloVe embeddings, we first obtained a noise estimate by evaluating the similarity between each of our 20 sampled enwik8 embeddings and the original GloVe embedding. To reiterate, the text in these corpora is comparable in nature to the text within the training data for the original GloVe embeddings.

The enwik8 sample corpora demonstrate the challenge of comparing the embeddings derived from corpora of different sizes: even when the corpus is included in the training set of a larger embedding, as is the case for samples from the enwiki8 corpora and the pre-trained GloVe embeddings, the similarity scores are relatively low (see Figure S5). Against this backdrop, the child-directed embeddings exhibit the similarity performance one would expect given their extreme small size relative to the original GloVe embeddings.

Category analysis. To obtain the semantic categories of concepts, we started with the categorisations available in the WordBank dataset (e.g Food & Drink, Clothing, Animals), and added some additional categories to cover concepts which were not included in the AoA set but existed in the word/image embedding intersection (e.g Weapons, Medical, Tools). Upon the addition of these new categories, the early-acquired concepts were reviewed, and some items were re-assigned to these new categories if appropriate. Category distributions are given in Table S8.

Hypernymy across agent types. Hypernymy is a measure of the superordinacy of a word. Our hypernymy values are obtained from WordNet (3) via the `NLTK` package in Python, and counted as the number of hypernyms a word has (i.e how many words are above it in the WordNet hierarchy). Therefore, lower hypernymy equates to higher superordinacy of a concept (i.e, more generality).

Examining the hypernymy of concepts acquired over time, we find that the structure-based concepts have lower hypernymy (higher generality) than the concepts in the control or AoA conditions to begin with, but increase in hypernymy over time. This is shown in Figure S14, with β coefficients for the fitted linear regressions shown in Table S9.

As expected, control sequences do not fluctuate in hypernymy over time. AoA agents begin by acquiring more specific concepts, and over time begin to learn more general terms. Both structural agents behave similarly to the control agent in terms of hypernymy: the hypernymy of their acquired concepts does not change over time.

Calculating influence of learned variables on concept selection. While all features are scaled to fall between 0 and 1, this scaling is generally based on the theoretical minima and maxima of the feature values. Consequently, there is still variation in the regions of the parameter space which is typically occupied by concepts in a knowledge state. This means that learned parameter values and weights are not directly comparable without some transformation.

[*] Obtained from http://mattmahoney.net/dc/textdata.html

To interpret the learned feature values and weights, we take a sample of 900 knowledge states (100 for each month) generated via random sampling as in the control agent. For each knowledge state, we obtain values of the parameters of interest and calculate the mean distance of each parameter's value from the target variable value obtained through training. This gives us a distribution of values for the distance from targets for each variable. We then multiply this mean distance by the learned weight for the variable. This provides a representative metric for learned variable importance.

Corpus	Total words	Unique words	Total documents	Avg wds per document
CHILDES	4.7×10^6	$12,252$	$5,360$	876
enwik8	1.2×10^7	$61,809$	$8,354$	$1,387$
GloVe (Wikipedia + GigaWord)	6.0×10^9	$400,000$	-	-

Table S1. Corpus sizes for child-directed speech corpus (from CHILDES), enwik8 and the original corpus used to train GloVe.

	Control		AoA	
Month (m)	t-statistic	p	t-statistic	p
16	91.70	$\ll 0.001^*$	125.77	$\ll 0.001^*$
17	90.24	$\ll 0.001^*$	118.79	$\ll 0.001^*$
18	112.70	$\ll 0.001^*$	142.24	$\ll 0.001^*$
19	108.43	$\ll 0.001^*$	134.30	$\ll 0.001^*$
20	117.57	$\ll 0.001^*$	131.31	$\ll 0.001^*$
21	111.16	$\ll 0.001^*$	143.14	$\ll 0.001^*$
22	113.86	$\ll 0.001^*$	150.17	$\ll 0.001^*$
23	122.33	$\ll 0.001^*$	133.76	$\ll 0.001^*$
24	118.89	$\ll 0.001^*$	131.83	$\ll 0.001^*$

Table S2. One sample t-test results for the comparison of control and AoA forced-choice results to chance performance (50% accuracy). At $\alpha = 0.05$, Bonferroni corrected for 18 individual comparisons to give adjusted threshold 0.0027, all comparisons are highly significantly different from chance performance in the forced choice task.

Predictor	df	F	p	η_p^2
Agent	$(1, 198)$	347.48	$< .001*$	0.627
Probe	$(1, 198)$	529.96	$< .001*$	0.719
Agent * Probe	$(1, 198)$	11.83	$< .001*$	0.069
Month	$(8, 1584)$	62.11	$.001*$	0.235
Agent * Month	$(8, 1584)$	2.61	$.008*$	0.011
Probe * Month	$(8, 1584)$	1.84	$.066$	0.008
Probe * Agent * Month	$(8, 1584)$	1.09	$.369$	0.005

Table S3. Repeated-measures ANOVA results for probe pair experiment with pre-trained GloVe word embeddings. Agent condition (AoA vs. control) was a between-subject factor, and probe condition (AoA-constrained vs. Unconstrained) and month were within-subject factors. $*$ indicates statistically significant results for α=0.05. df = degrees of freedom; η_p^2 is partial η^2 effect size.

Predictor	df	F	p	η_p^2
Agent	$(1, 198)$	1165.70	$< .001*$	0.850
Probe	$(1, 198)$	12.03	$< .001*$	0.070
Agent * Probe	$(1, 198)$	6.90	$.009*$	0.049
Month	$(8, 1584)$	25.50	$< .001*$	0.111
Agent * Month	$(8, 1584)$	4.55	$< .001*$	0.020
Probe * Month	$(8, 1584)$	1.65	0.107	0.007
Probe * Agent * Month	$(8, 1584)$	0.49	0.863	0.002

Table S4. Repeated-measures ANOVA results for probe pair experiment with word embeddings derived from CHILDES dataset of child-directed speech. Agent condition (AoA vs. control) was a between-subject factor, and probe condition (AoA-constrained vs. Unconstrained) and month were within-subject factors. $*$ indicates statistically significant results for α=0.05. df = degrees of freedom; η_p^2 is partial η^2 effect size.

Predictor	df	F	p	η_p^2
Agent	$(1, 198)$	49.27	$< .001*$	0.200
Probe	$(1, 198)$	0.31	0.577	0.015
Agent * Probe	$(1, 198)$	17.46	$< .001*$	0.092
Month	$(8, 1584)$	112.98	$< .001*$	0.059
Agent * Month	$(8, 1584)$	1.45	0.169	0.006
Probe * Month	$(8, 1584)$	1.10	0.364	0.005
Probe * Agent * Month	$(8, 1584)$	1.04	0.401	0.004

Table S5. Repeated-measures ANOVA results for probe pair experiment with word embeddings derived from the enwik8 Wikipedia dataset. Agent condition (AoA vs. control) was a between-subject factor, and probe condition (AoA-constrained vs. Unconstrained) and month were within-subject factors. $*$ indicates statistically significant results for α=0.05. df = degrees of freedom; η_p^2 is partial η^2 effect size.

Feature	Description	
Node measures	Distance in full space (mean/max/min)	The mean/max/min magnitude of a concept's distances from other concepts in the full concept space.
	Distance within knowledge state (mean/max/min)	The mean/max/min magnitude of a concept's distances from concepts in the existing knowledge state
	Degree in full space (k_{full})	Number of vertices between a concept and all other concepts in the full space.
	Degree in knowledge state ($k_{\text{knowledge}}$)	Number of vertices between a concept and all concepts in the existing knowledge state.
	Betweenness in full space	Fraction of shortest paths in the full space graph which pass through the concept.
	Betweenness in knowledge state	Fraction of shortest paths in the knowledge state graph which pass through the concept.
	Clustering in full space	The fraction of possible triangles that pass through the concept in the full space which are realised.
	Clustering in knowledge state	The fraction of possible triangles that pass through the concept in the knowledge state which are realised.
Knowledge state measures	Average dimension coverage	The average proportion of embedding dimensions' overall variability in the full concept set which is covered by the concepts in the knowledge state
	Degree distribution skew (proxy)	The skew of the degree distribution of the knowledge state. Skew is approximated as $\frac{\text{max}(k)-\text{mean}(k)}{\text{max}(k)-\text{min}(k)}$

Table S6. Features tested for knowledge state classification

	AoA probe						Control probe					
	A-M v T-O		A-M v AoA		T-O v AoA		A-M v T-O		A-M v AoA		T-O v AoA	
Month	t	p-value	t	p-value	t	p-value	t	p-value	t	p-value	t	p-value
16	4.07	$\ll 0.001^*$	3.64	$\ll 0.001^*$	1.02	0.816	8.78	$\ll 0.001^*$	8.42	$\ll 0.001^*$	0.87	0.385
17	4.54	$\ll 0.001^*$	3.70	$\ll 0.001^*$	1.47	0.247	8.63	$\ll 0.001^*$	6.53	$\ll 0.001^*$	2.43	0.016
18	5.04	$\ll 0.001^*$	2.40	0.018	3.05	0.012	9.71	$\ll 0.001^*$	7.54	$\ll 0.001^*$	2.17	0.032
19	3.14	0.002	0.47	0.637	3.13	$\ll 0.001^*$	5.15	$\ll 0.001^*$	2.19	0.030	3.19	0.002
20	2.64	0.009	1.16	0.249	3.84	$\ll 0.001^*$	5.58	$\ll 0.001^*$	3.00	0.003	2.61	0.010
21	3.01	0.003	0.37	0.715	3.79	$< 0.001^*$	5.62	$\ll 0.001^*$	3.07	0.002	2.66	0.008
22	1.87	0.062	3.10	0.002	5.39	$\ll 0.001^*$	6.12	$\ll 0.001^*$	2.82	0.005	3.37	$\ll 0.001^*$
23	0.57	0.573	4.05	$\ll 0.001^*$	3.51	$< 0.001^*$	3.59	$< 0.001^*$	0.17	0.868	3.47	$\ll 0.001^*$
24	1.44	0.152	3.95	$\ll 0.001^*$	2.53	0.014	2.70	0.001	0.02	0.984	2.78	0.006

Table S7. Results for monthwise pairwise t-tests for forced-choice performance between each pair of model types. A-MAoA-Matched; T-O=Task-Optimised For $\alpha = 0.05$, Bonferroni corrected threshold is $0.05/54 = 0.0009$

Category	Non-AoA	AoA	Total
Animals	64 (0.218)	34 (0.241)	98 (0.226)
Body parts	2 (0.007)	0 (0.000)	2 (0.005)
Clothing	19 (0.065)	17 (0.122)	36 (0.083)
Food/Drink	42 (0.143)	28 (0.199)	70 (0.161)
Furniture/Rooms	11 (0.038)	17 (0.121)	28 (0.065)
Household	51 (0.174)	19 (0.135)	70 (0.161)
Medical	6 (0.020)	0 (0.000)	6 (0.014)
Music	20 (0.068)	0 (0.000)	20 (0.046)
Outside	12 (0.041)	5 (0.035)	17 (0.039)
People	0 (0.000)	5 (0.035)	5 (0.012)
Places	5 (0.017)	1 (0.007)	6 (0.014)
Sports/Activities	24 (0.082)	0 (0.000)	24 (0.055)
Tools	6 (0.020)	2 (0.014)	8 (0.018)
Toys	1 (0.003)	4 (0.028)	5 (0.012)
Vehicles	17 (0.058)	9 (0.064)	26 (0.060)
Weapons	7 (0.024)	0 (0.000)	7 (0.016)
Other	6 (0.020)	0 (0.000)	6 (0.014)
Total	293 (1.000)	141 (1.000)	434 (1.000)

Table S8. Frequencies of each semantic category within each concept set. Proportions in brackets represent the proportion of the concept set which is comprised of concepts from a given semantic category.

Condition	R^2	F	p	β_{month}	CI	t
Control	< 0.01	2.154	0.14	0.014	$[-0.004, 0.027]$	1.468
AoA	0.01	21.940	$\ll 0.001*$	-0.037	$[-0.053, -0.022]$	4.684
Structural-AoA-Matched	< 0.01	2.460	0.117	0.023	$[-0.006, 0.051]$	1.568
Structural-Task-Optimised	< 0.01	0.190	0.663	-0.005	$[-0.029, 0.019]$	0.435

Table S9. β **weights for gradient of hypernymy over time. * indicates a statistically significant trend at** α**=0.05**

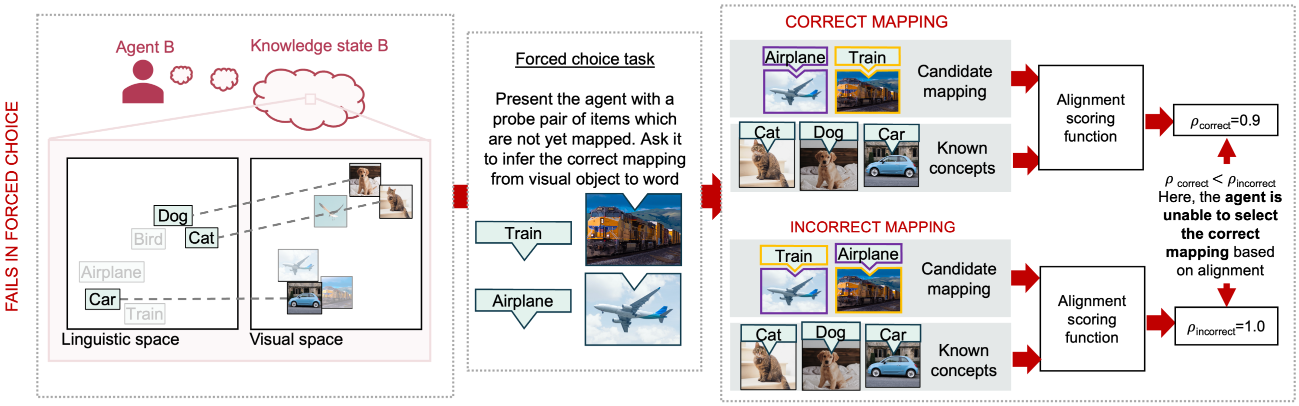

Fig. S1. An example of an Agent failing the forced choice task presented in Figure 4.

References

1. B MacWhinney, The childes project: Tools for analyzing talk: Volume i: Transcription format and programs, volume ii: The database (2000).
2. M Antoniak, D Mimno, Evaluating the stability of embedding-based word similarities. *Transactions Assoc. for Comput. Linguist.* **6**, 107–119 (2018).
3. GA Miller, Wordnet: a lexical database for english. *Commun. ACM* **38**, 39–41 (1995).

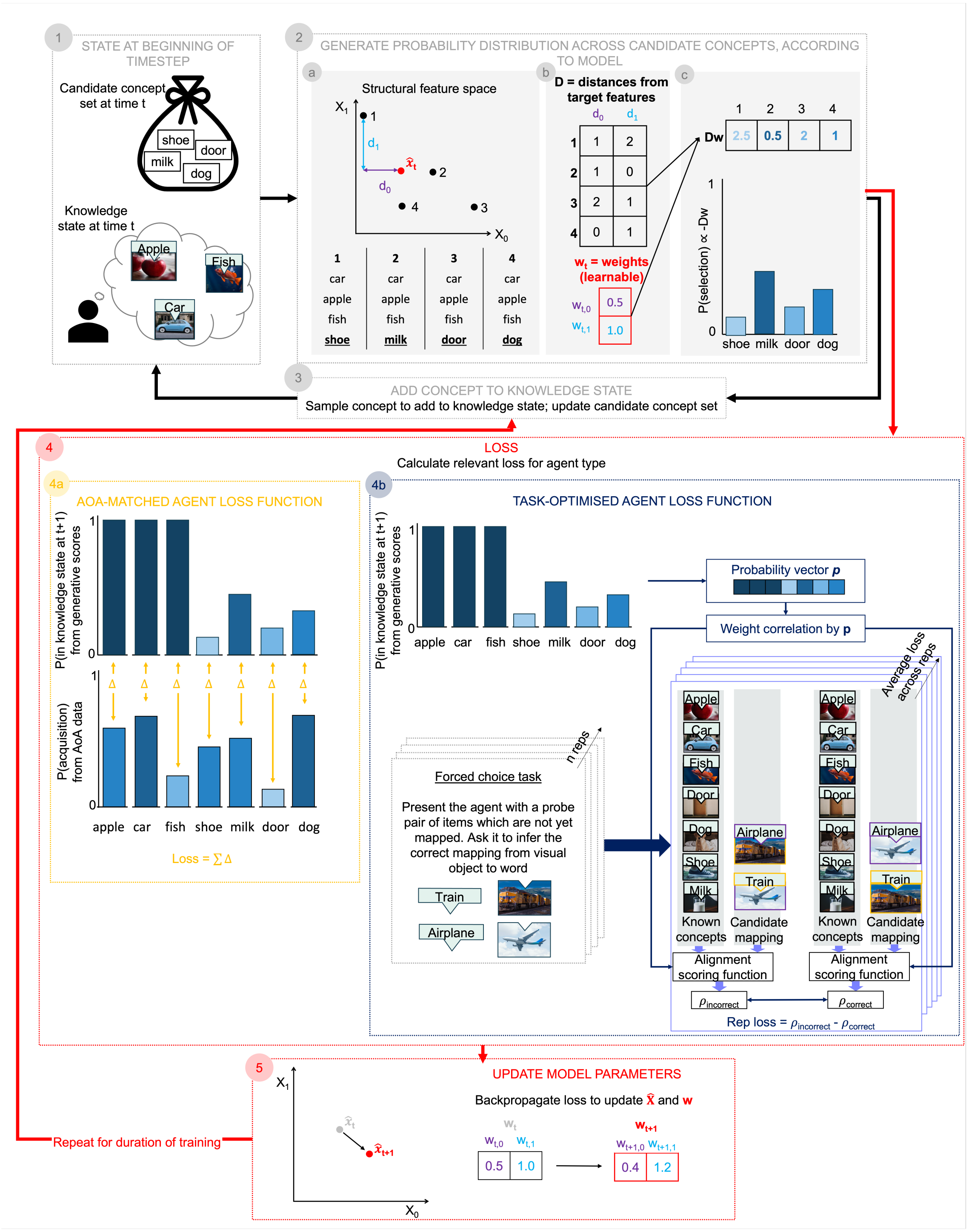

Fig. S2. Diagram showing the training and generative processes of the structural agents. Model parameter training proceeds as follows: assume we start observing training when the knowledge state and candidate concept set are as shown in panel 1. Here, the agent knows {apple, car, fish}. Panel 2 shows how the agent uses its internal model to calculate a probability distribution for the selection of the next concept from the n_c candidates shown in panel 1. The internal model consists of target structural feature vector $\hat{\mathbf{x}}_t \in \mathbb{R}^k$ and weight vector $\mathbf{w}_t \in \mathbb{R}^k$. For visualisation purposes we show the case of $k = 2$, but in the main study $k = 7$ (as 7 structural features were identified in the structural analysis). Internal model parameters and model training steps are highlighted in red. In panel 2a, all structural features are calculated for each candidate knowledge state (where, for example, the candidate knowledge state associated with acquiring the concept 'shoe' is {apple, car, fish, shoe}). Then, the distance of each candidate knowledge state from $\hat{\mathbf{x}}_t$ in each dimension is calculated, yielding distance matrix $\mathbf{D}^{n_c \times k}$, shown in panel 2b. The vector of weighted distances from target in each dimension, **Dw**, is calculated in panel 2c for all candidate knowledge states. This is transformed into a probability distribution across candidate concepts, where candidate knowledge states with features close to $\hat{\mathbf{x}}_t$ are chosen with higher probability. In step 3, the next concept for the knowledge state is sampled from this distribution. In model training, the agent progresses to step 4 at the end of each month m, where it calculates the relevant loss for optimisation. For the AoA-matched agent (shown in orange, 4a), the loss is the distance between the expected probability that each concept will be in the knowledge state according to the model, and the probability of each concept being acquired by the end of month m in the AoA data. In this instance, the acquisition of each concept is modelled as an independent Bernoulli random variable. For the Task-optimised agent (shown in blue, 4b), the loss is $\rho_{\text{incorrect}} - \rho_{\text{correct}}$, averaged across a series of forced choice tasks. Crucially here, the correlations are weighted by the probability that each concept will be selected for the knowledge state. Then, in step 5, the loss term is backpropagated to update parameters $\hat{\mathbf{x}}$ and $\mathbf{w}$. Once a model is trained, parameters are fixed and knowledge state trajectories are generated by repeating steps 1, 2 and 3.

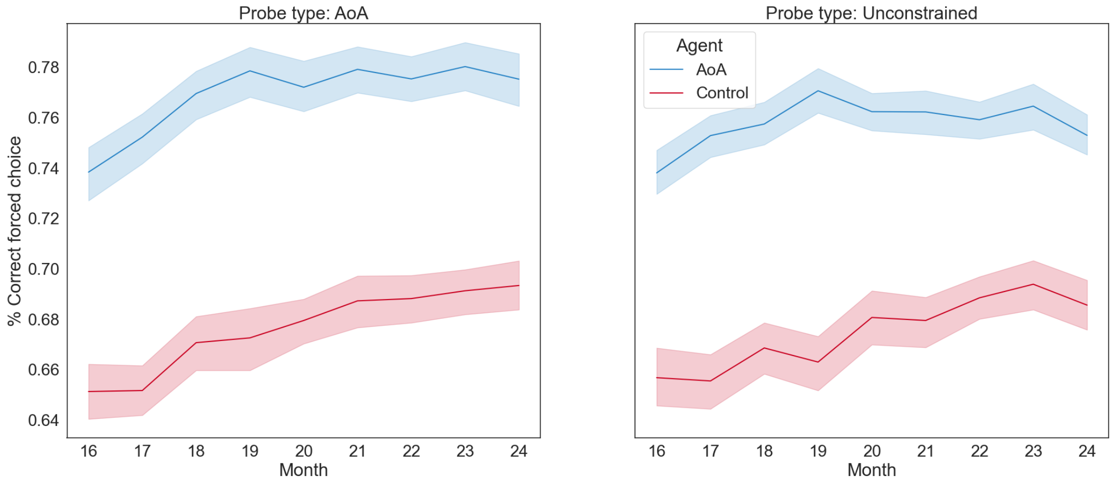

Fig. S3. Forced choice results where linguistic embeddings are derived from child-directed speech corpus CHILDES.

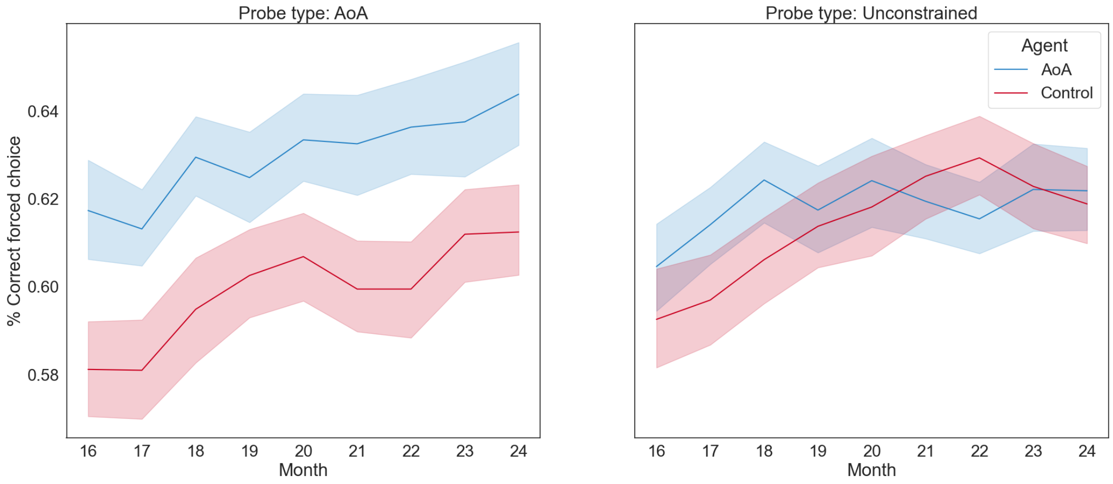

Fig. S4. Forced choice results where linguistic embeddings are derived from a subset of the enwik8 corpus, with the same size as the child-directed speech corpus CHILDES.

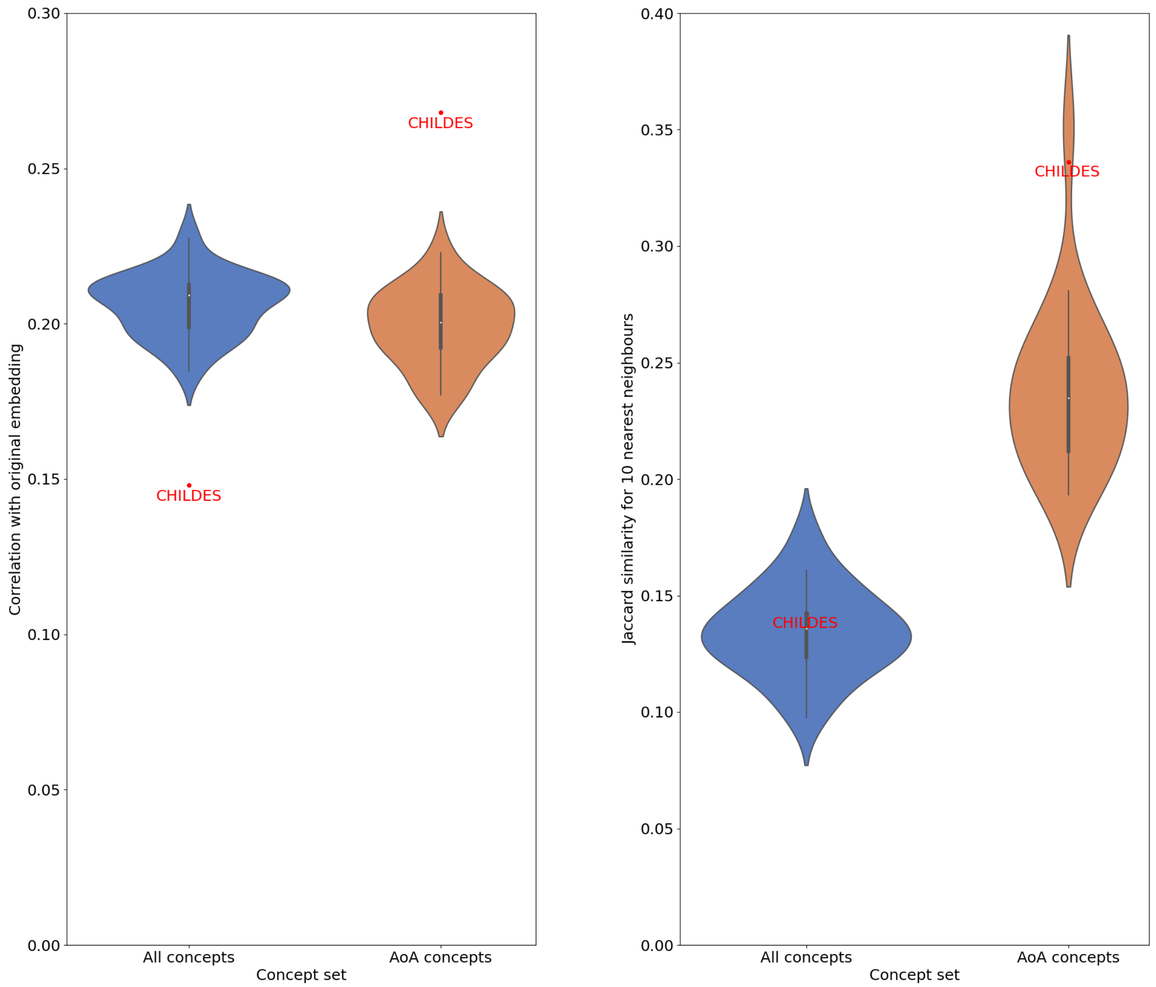

Fig. S5. (a) Correlations of pairwise relationships and (b) Jaccard similarity scores for 10-nearest-neighbours of concepts across 20 embeddings of comparable size to the CHILDES corpus, sampled from the enwik8 corpus. The corresponding performances of CHILDES on the relevant metrics are also shown. Performance is shown for all concepts in the set (blue) and for early-acquired concepts only (orange). On both similarity measures, the CHILDES embeddings perform comparably to the comparably-sized samples from the GloVe embedding training corpus. CHILDES embeddings even outperform the enwik8 embeddings of comparable size for early-acquired words. This shows that the CHILDES embeddings are as similar to the large-scale pre-trained GloVe emebeddings as they could be expected to be, given their corpus size. Thus, there is no evidence here to suggest that the similarity relationships for children are substantially different to those for adults, for the concepts used in this contribution.

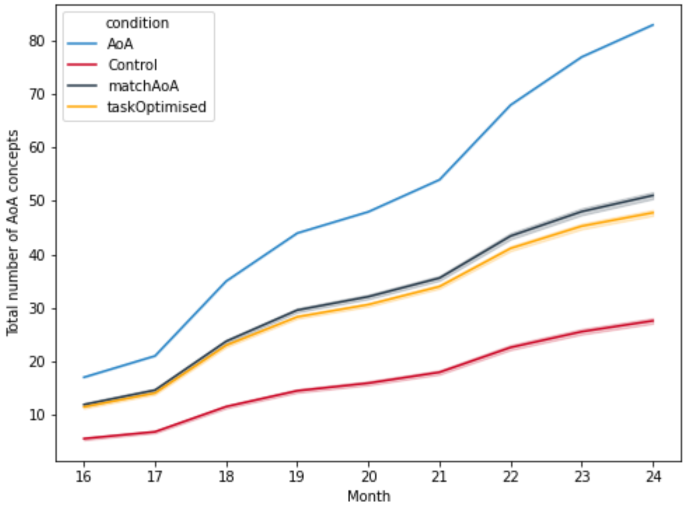

Fig. S6. Number of concepts acquired which are AoA, by generative condition. The AoA line represents all items in the sample being AoA.

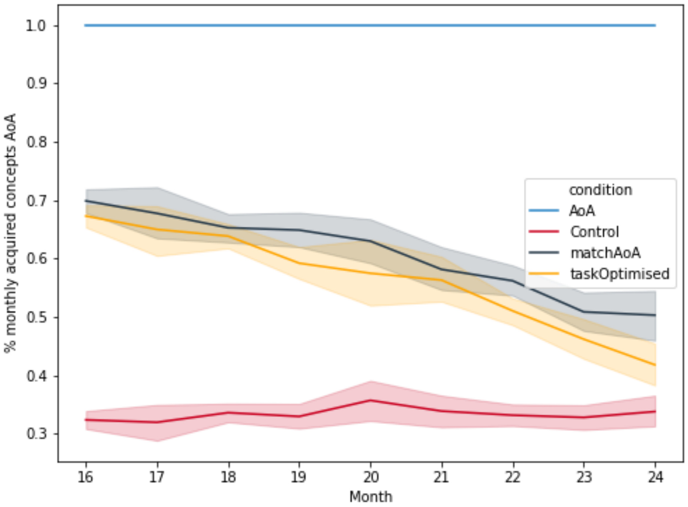

Fig. S7. Proportion of concepts acquired in each month which are AoA.

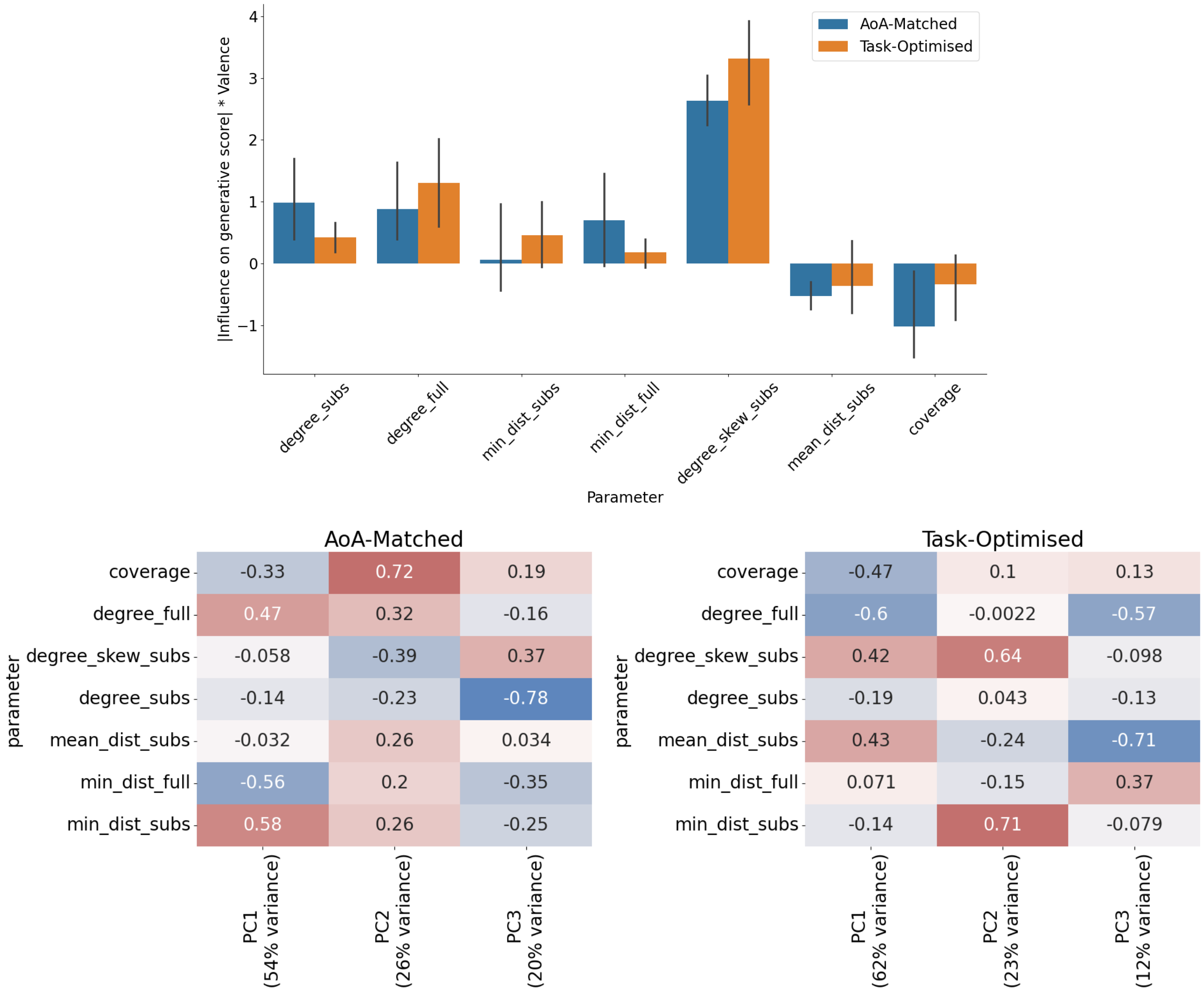

Fig. S8. (A) Mean learned importances of features for selecting new concepts to add to the knowledge state, for each generative agent type. Error bars represent 95% confidence intervals across restarts. (B) Variable loadings on principal components of variation in absolute influence scores across restarts for generative models. For both model types, over 80% of variance is captured by the first two principal components. For the AoA-Matched agent, the first principal component is dominated by an antagonistic relationship between the minimum distance within full space and the minimum distance within the knowledge state and the degree in full space; for the optimal agent, the first principal component is dominated by an antagonistic relationship of the degree in full space and average dimensional coverage of the knowledge state with the mean distance in the knowledge state and the degree skew in the knowledge state.

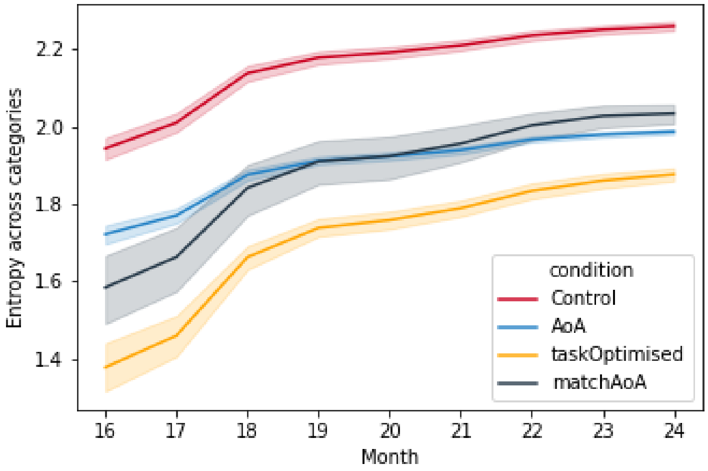

Fig. S9. Overall entropy of knowledge state's category distribution after each month of concept acquisition. Shaded areas represent 95% confidence intervals around the mean, based on 100 simulated agents per condition.

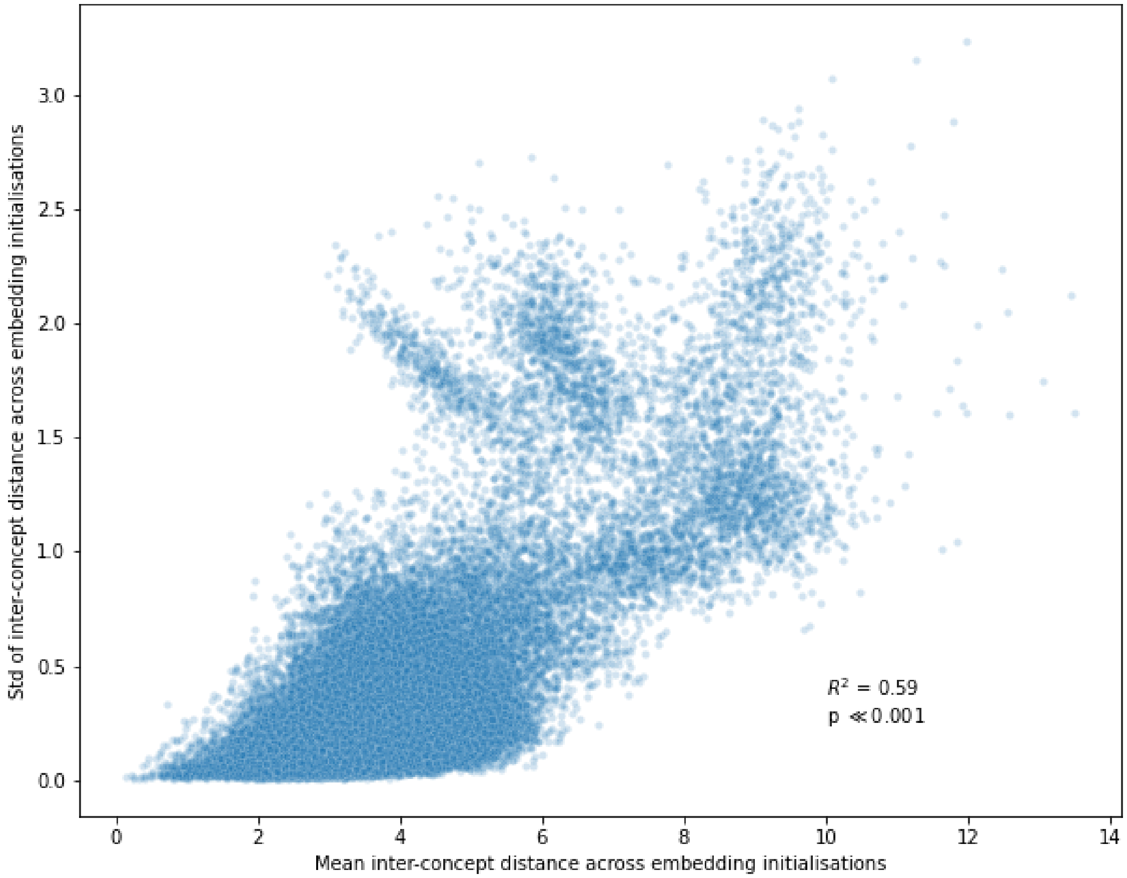

Fig. S10. Relationship between the average distance between two concepts and the standard deviation of the relationship across multiple initialisations of the image embedding.

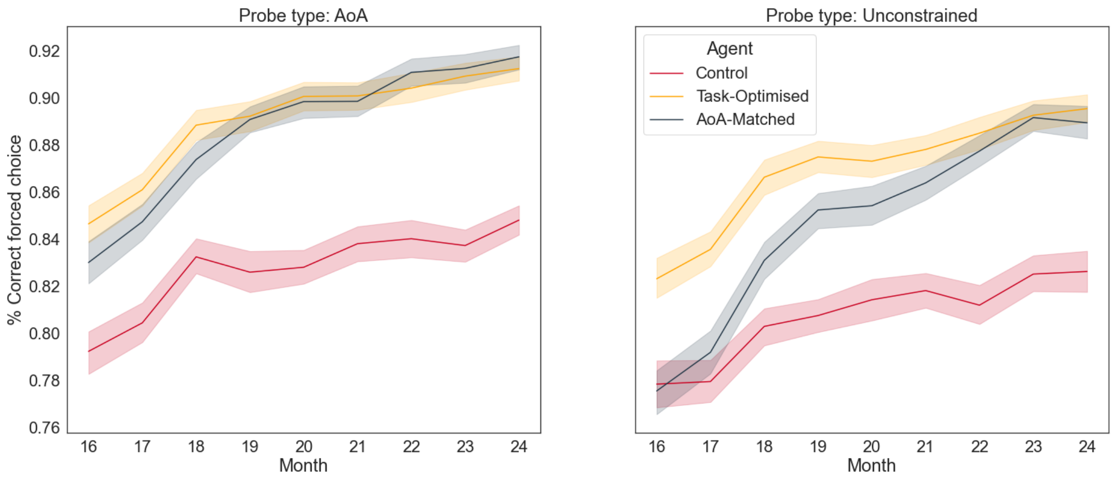

Fig. S11. Forced choice performance for control, AoA-Matched and Task-Optimised agents, when knowledge states are not permitted to contain any early-acquired concepts.

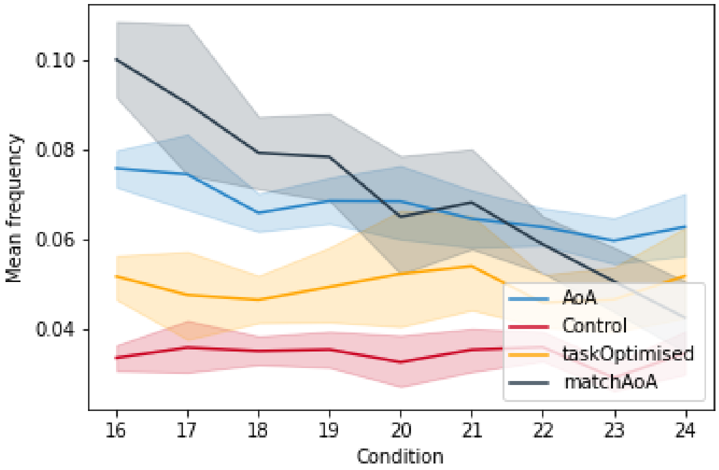

Fig. S12. Frequency of concepts in knowledge states of different agent types over time, when early-acquired concepts are permitted for inclusion in knowledge states.

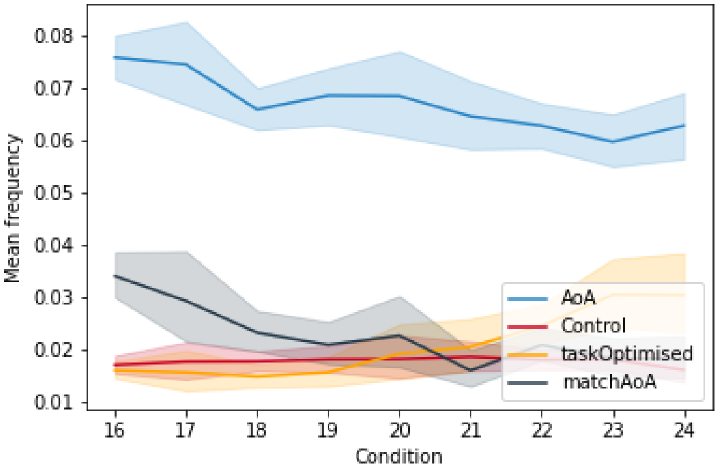

Fig. S13. Frequency of concepts in knowledge states of different agent types over time, when early-acquired concepts are excluded from knowledge states.

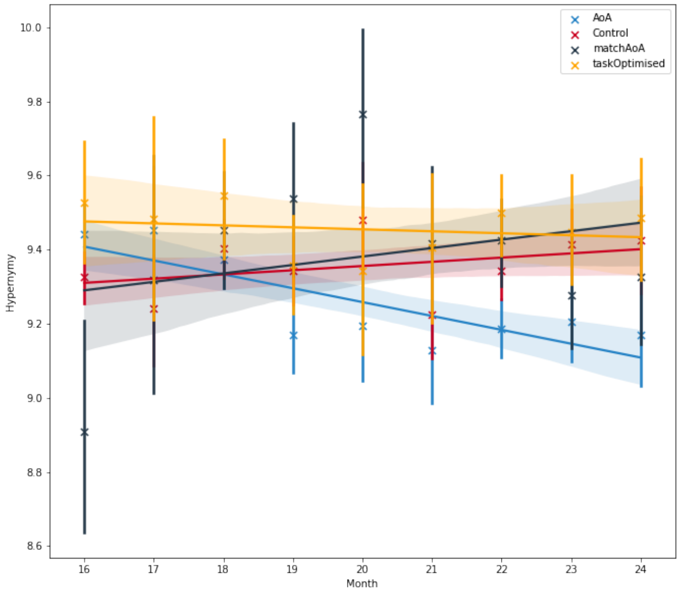

Fig. S14. Hypernymy of acquired concepts across months by condition.
